# Nanotechnology-Driven Treatment Strategies for Breast Cancer: Recent Advances and Innovations

**DOI:** 10.32604/or.2025.066624

**Published:** 2025-09-26

**Authors:** Neha Raina, Radha Rani, Mahika Kanojia, Avril Mathias, Keshav Raj Paudel, Ashish Garg, Hardeep Singh Tuli, A. T. M. Mijanur Rahman, Vetriselvan Subramaniyan, Madhu Gupta

**Affiliations:** 1 Centre for Global Health Research, Saveetha Medical College and Hospitals, Saveetha Institute of Medical and Technical Sciences, Chennai, 602105, India; 2 Department of Pharmaceutics, School of Pharmaceutical Sciences, Delhi Pharmaceutical Sciences and Research University (DPSRU), New Delhi, 110017, India; 3 Department of Pharmaceutics, Nitte College of Pharmaceutical Sciences, Bangalore, 560064, India; 4 Centre for Inflammation, Faculty of Science, School of Life Sciences, Centenary Institute and University of Technology Sydney, Sydney, NSW 2050, Australia; 5 Department of Pharmaceutics, Guru Ramdas Khalsa Institute of Science and Technology, Jabalpur, 483001, India; 6 Centre of Excellence in Computational Research and Drug Discovery, Department of Bio-Sciences and Technology, Maharishi Markandeshwar Engineering College, Maharishi Markandeshwar (Deemed to Be University), Mullana, Ambala, India; 7 Bio-nanotechnology and Nanomedicine Lab., Dept. of Applied Nutrition and Food Technology, Islamic University, Kushtia, 7003, Bangladesh; 8 Division of Pharmacology, Faculty of Medical and Life Sciences, Sunway University, Bandar Sunway, 47500, Malaysia

**Keywords:** Breast cancer, diagnosis, therapeutic strategy, nanotechnology, clinical trials

## Abstract

Breast cancer is among the most prevalent cancers in females globally and has the highest mortality rate. The emergence of pharmacologic resistance in breast cancer is a significant challenge for researchers in the pursuit of effective treatment. Investigations in cancer nanotechnology have been transformed by the advancement of smart polymers, lipids, and inorganic materials. Research is now being conducted in the field of innovative nano-pharmaceutical formulations aimed at enhancing the efficacy and durability of chemotherapy. Nanotechnology-based delivery systems are beneficial for combating breast cancer due to theranostic applications, augmented drug encapsulation, decreased degradation, and minimal adverse effects. This review discusses breast cancer and its stages, various risk factors, and pathogenesis, in addition to diagnosis and treatment. Novel nanocarriers are included with the most recent findings in this area and the potential use of these nanocarriers in cancer therapy that centers on their clinical usage for improved treatment. Patents and promising clinical trial results are also explained in detail, with nanotoxicity, ethical concerns, and regulations. This study underscores the importance of treatment strategies using nanotechnology, highlighting the advancing paradigm of breast cancer care. This article examines the prospects, obstacles, and future trajectories of nanomedicines.

## 1 Introduction

Cancer represents a proliferative disorder with a multifaceted character and is considered the leading cause of significant demises worldwide [1]. Now, it is regarded as the most common barrier to life, shortening the life span worldwide [2]. Mutational changes in the genetic material will result in uncontrolled growth of cells and tissue due to malfunction and metastasis to different body parts [3]. Among all, Breast cancer (BC) is responsible for the maximum number of fatalities in females [4]. The increasing rate of death of females due to BC makes it the second-highest form of cancer, giving more importance to its cure [5]. Among women, annually, BC is the leading cause of mortality [6], around 2.5%, corresponding to a 1 in 39 chance of death [7,8]. Furthermore, according to the reports of 2020 Globocan data, BC recorded 10.6% of cancer-related fatalities, covering almost 13.5% of all cancer diagnoses in India, with a cumulative risk of 2.81 [9]. The prevalence of BC is higher in developed nations with high Human Development Index (HDI) and transitioning republics.

**Figure 1:**
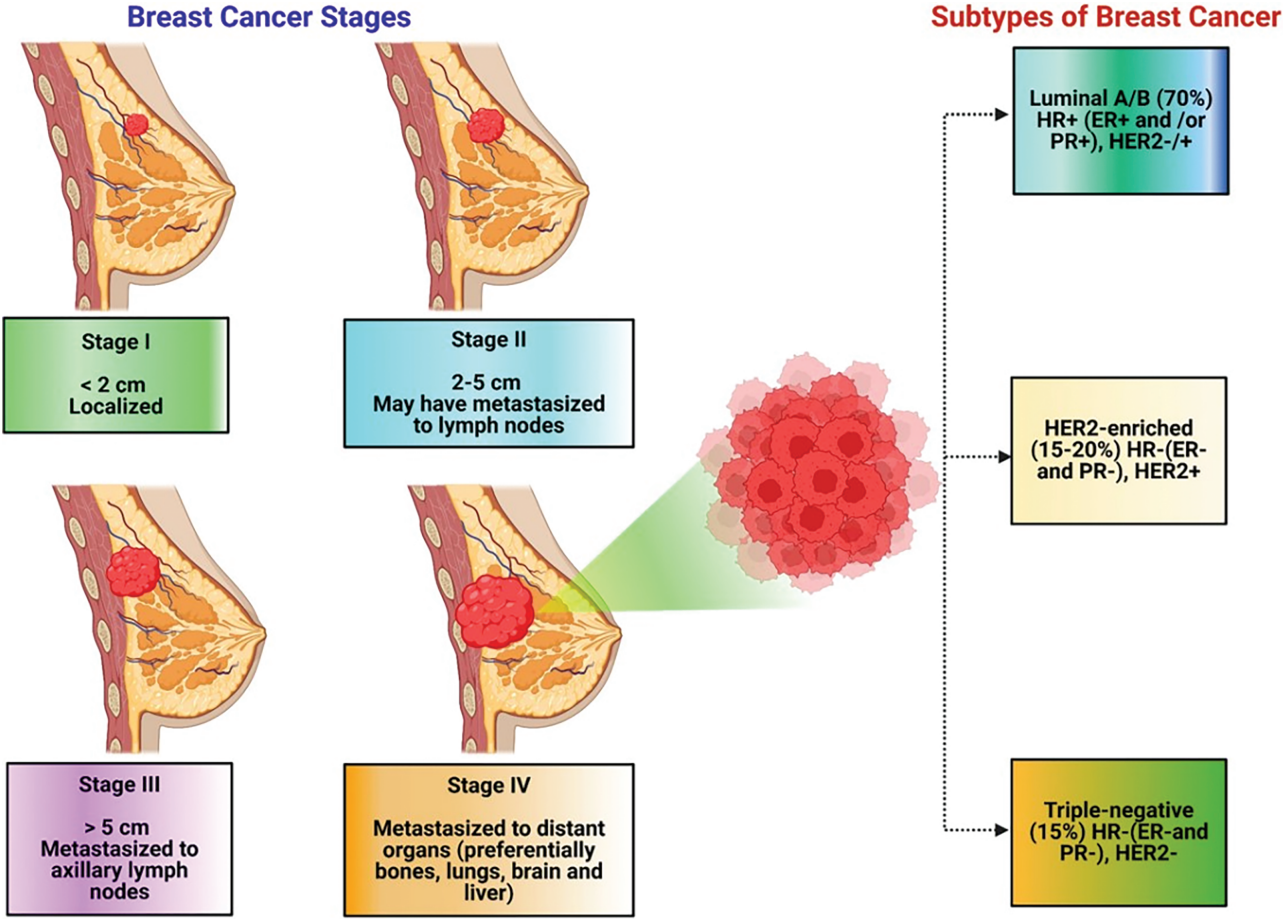
Clinical classification of breast cancer

It is categorized into four stages (from stage 0 to stage 4) depending upon the size and type of the tumor (Fig. 1), as well as the penetrability of tumor cells within breast tissues [10–12]. BC is primarily categorized into two main types: invasive or non-invasive breast cancer. By the histopathological or biochemical assessment of BC cases, invasive BC (IBC) may be categorized into three primary subtypes, namely Luminal, Non-Luminal (human epidermal growth factor receptor 2 (HER2)+), and Triple-negative BC (TNBC). Luminal BC refers to a subtype of IBC that exhibits hormone receptor expression. This kind of IBC sometimes presents in histological forms, including mucinous, invasive lobular, tubular, invasive cribriform, etc. [13–15]. Furthermore, based on the proliferation pathway and luminal regulation, luminal BC is further classified into subtypes A and B [16,17]. TNBC is a highly aggressive form of breast cancer, and its prevalence continuously increases. It is associated with the worst prognosis and significantly decreased patient life span compared to other types of breast cancer [11,12]. Population- and epidemiology-based studies prove that TNBC mainly occurs in women under the age of 40 and accounts for approximately 25% of all breast cancer diagnoses [16]. The mortality rate among individuals with TNBC approaches 40% during the first five years following diagnosis. The mortality rate during the third month following TNBC recurrence may reach up to 75%, reflecting the disease’s aggressive and refractory nature [17]. TNBC is characterized by a lack of useful therapy options. This is largely due to the absence of estrogen receptor (ER), progesterone receptor (PR), and human epidermal growth factor receptor 2 (HER2) expression, making endocrine and targeted therapy useless. Therefore, chemotherapy remains the main treatment method for dealing with TNBC [18,19]. Over the past few years, nanotechnology has offered several advantages in the treatment of breast cancer or TNBC, addressing key challenges such as drug resistance, metastasis, and toxicity. One of its primary benefits is enhanced targeted drug delivery, where nanocarriers enable precise accumulation of therapeutic agents in TNBC cells while minimizing side effects on healthy tissues [20]. Nanotechnology also plays a crucial role in overcoming drug resistance by bypassing efflux pumps, ensuring better intracellular drug retention, and improving treatment efficacy. These advancements demonstrate how nanotechnology is revolutionizing TNBC treatment by offering more precise, effective, and less toxic therapeutic options [21,22].

This paper critically reviews the recent developments in nanocarriers for breast cancer. This detailed literature review discusses the stages of breast cancer, pathogenesis, various risk factors with diagnosis, nanocarrier-based treatment approaches, and the most recent findings in this area, with their limitations, nanotoxicity, and ethics are highlighted in the manuscript. Patents and promising clinical trials and regulatory aspects are stated in this review paper, along with prospects. This study underscores the importance of treatment strategies using nanotechnology, highlighting the advancing paradigm of breast cancer care.

## 2 Breast Cancer: Pathogenesis, Diagnosis and Risk Factors

The essential clinical factors promoting the progression of BC comprise high hormonal status (ER, PR & HER2), cellular factors, microRNAs (miRNAs), and specific gene mutations [23]. BC Stem Cells (BCSCs) are considered central cancer-driving cells. BCSCs are responsible for tumor initiation and facilitate therapeutic resistance and tumor malignancy. Highly active cytokines like Transforming growth factor beta receptor (TGFBR), Chemokines, and interleukins contribute to their role in the onset of BC malignancy. Approximately fifty distinct chemokines mainly induce chemotaxis of immune cells via twenty-five distinct seven-transmembrane G-protein coupled receptors. Plasma levels of IL8 and IL6 (also called CXCL8) correlate with the stage and mortality of BC and the resistance to chemotherapeutics [24]. Xenobiotic chemicals target breast tissue and generate reactive free radicals, causing breast cancer. These carcinogenic chemicals are metabolism-resistant, fat-soluble, and stored in breast adipose tissue [24]. CG protein-coupled receptor, e.g., Ca^2+^-sensing receptor (CaSR), gained attention as an oncogene and acts as an oncoprotein in the case of BC following skeletal metastases pathogenic pathway [25].

Cancer development is facilitated by oncogenic miRNAs, which impede tumor suppressor gene expression. Conversely, some miRNAs can downregulate oncogenic genes, demonstrating tumor suppressor characteristics [26]. Biomarkers are significant in managing BC therapy as they predict treatment outcomes and also have great importance in the early diagnosis of breast carcinoma [27]. Different types of biomarkers used for the prognosis of BC comprise BRCA1/2 gene mutations, serum biomarkers like carcinoembryonic antigen (CEA) or cancer antigen 15-3 (CA 15-3), circulating tumor-derived DNA, circulating tumor cells, and a multi-analyte profile of Oncotype DX or uPA/PAI-1, etc. [28]. Exosomes with various tumor antigens have become a great hotspot in diagnosis because of the elevated secretion level on the cancer cell’s surface. Furthermore, Aptamer probe hybridization (APH) and fluorescence *in situ* hybridization (FISH) are the two methods in nucleic acid hybridization that can identify unique tumor biomarker fragments and look for novel tumor biomarkers. CA125, HER-2, CA153, and CEA are partial oncogene proteins related to BC, which are used primarily as predictive markers of BC detection [29,30].

Mucinous carcinoma represents a distinct and infrequent variant of breast cancer, accounting for approximately 2% of the overall incidence of this disease [31]. The differential expression of mucins in BC has been investigated to explore its potential diagnostic and prognostic significance. The expression of MUC1 is notably increased in around 59% of healthy breast tissues, and this elevated level of expression is likewise observed in the malignant ducts near the normal tissue. As compared to normal breast epithelium, BC tissues are diagnosed with upregulated secretion and membrane-bound mucins in ductal adenocarcinoma of the breast [32,33]. Combining the currently employed blood tests and scanning procedures with novel nanoparticle-based techniques has been anticipated to improve the diagnosis [34].

Diagnostic imaging and image-guided needle biopsies are integral components in diagnosing, planning treatment, and determining the stage of BC in patients. The mammograms are conducted to detect a mass and those with a documented history of BC within the last five years [35]. BC incidences become more common even after the research is carried out on a laboratory and commercial scale and remain an economic burden for women. The risk factors responsible for BC among women can be genetic or non-genetic [36]. The genetic background of a woman plays a significant role in determining her susceptibility to developing BC. Birth weight is a risk factor for BC [37]. Pathogenetic alterations in genes like “SNPs, CHEK2, BRCA1, and BRCA2” are at a high risk of inducing tumors and fall under the category of genetic factors [38]. Inherited alterations in the genes (BRCA1 & BRCA2) involved in the DNA strand repair caused approximately 2.5% of all BC incidences [39]. Other genes that rarely carry the mutations from parents to offspring are PTEN, TP53, STK11 or LKB1, CDH1, ataxia telangiectasia mutated, NBN, CHEK2, and BRCA2 [40].

Fibroadenoma (FA) is a prevalent lesion in the breast, accounting for 25% of women who do not exhibit any symptoms. FA is a collective term used to describe a cluster of hyperplastic breast lobules that are regarded as deviations from typical patterns of development and involution. Multiple studies have provided evidence suggesting that familial aggregation constitutes a genetic predisposition for developing breast cancer. FA exhibiting significant alterations is recognized as a precancerous lesion, with a relatively low incidence of malignant transformation ranging from 0.12% to 0.3%. The most common form of malignancy that arises from such lesions is lobular carcinoma *in situ* [41]. Non-genetic risk factors of BC are age, high mammary gland density of the breast, a higher index of body mass (BMI), personal pathological history of breast, exposure of chest to radiation like X-ray, exogenous intake of female hormones, reproductive factors, alcohol and insufficient physical activity [36]. The primary origin of estrogen in women after menopause arises from converting androstenedione to estrone by adipose tissue. Consequently, obesity is linked to a sustained elevation in estrogen exposure over an extended period [41]. BC is additionally linked to lifestyle factors. Based on existing evidence, there is a positive link between daily alcohol intake and the risk of developing BC. According to research, silicone implants may have a reduced likelihood of developing BC [42].

As per WHO, the breastfeeding rate is much lower than the recommended rate, which becomes the relative risk factor of BC incidents, especially in high-income countries. Breastfeeding for 12 months can effectively reduce the risk of BC incidences by up to 4% [43]. However, the mechanism of BC protection due to breastfeeding is not completely clear; still, it is believed that increased concentration of an oncogene named pregnancy-associated plasma protein A (PAPP-A) during the pregnancy period is inhibited due to increased stanniocalcin glycoproteins (STC1 and STC2) secretion in the lactation process. This PAPP oncogene, in case of low breast-feeding rate, accelerates the process of tumor formation [43]. Like age and BMI of women, mammographic density is taken into consideration as a non-genetic cancer-causing risk factor. Women with high mammographic density are more prone to BC risk as compared to women with low mammographic density. Men, in comparison to women, have a low mammographic density, so they are less prone to BC [44].

## 3 Treatment Strategies for Breast Cancer

**Figure 2:**
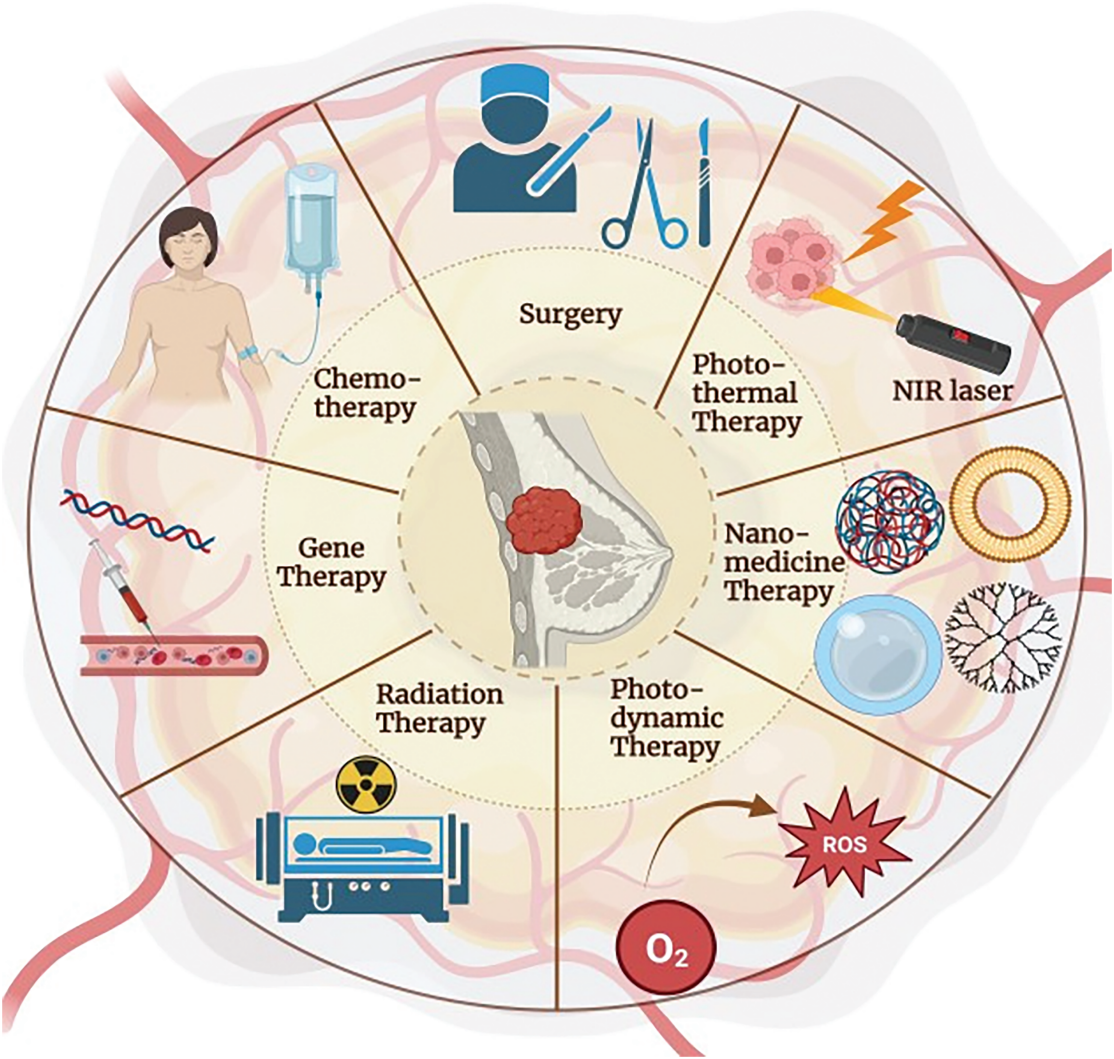
Treatment strategy for breast cancer

BC treatment includes immunotherapy, hormonal therapy, chemotherapy, radiation therapy, and surgery [45], where grade, molecular subtype, and stage of BC are the basis of BC management selection [46]. Currently, targeted treatment of BC aiming at HER2+ is the most commonly used therapy. Personalized treatment is used for treating metastatic breast cancer; however, it has no benefit in triple-negative BC and is fraught with problems due to drug resistance [47]. With time, the use of chemotherapy causes resistance and serious side effects like alopecia and a negative impact on healthy cells. So, to overcome this, radiation therapy, hormonal therapy, immunotherapy, and nanotechnology are highly emerging treatment methods for BC, either along with chemotherapy or alternative cytotoxic drugs. BC treatment strategies include endocrine therapy, chemotherapy, radiation, immunotherapy, and surgery (Fig. 2). Surgery is done when the tumor is in its initial stage of non-invasiveness and surgically removes the mass of tumor cells located in the breast and lymph nodes. Drugs used in conventional chemotherapy cause cell death in cancerous cells as well as normal and healthy cells. They also require high doses for their efficient therapeutic actions. Therefore, all conventionally used BC therapies involve several limitations [48].

BC involves a different pathogenesis type that requires selective treatment to avoid serious side effects. Newer therapies have evolved as a combined therapy of traditional chemotherapeutic drugs with monoclonal antibodies for treating BC, which provide satisfactory clinical outcomes by immune cell death (Table 1). The dose used is sufficient to trigger immune responses against the tumor and prevent it from destroying the immune response. Due to the dose limitation of traditional chemotherapy, new targeted therapies that target the signaling mechanism responsible for BC occurrence are discussed in this section. These signaling mechanisms include checkpoint inhibitors, microRNA, apoptotic cell death pathway, Poly (ADP-ribose) polymerase (PARP) inhibitors, mitogen activated protein kinase (MAPK) inhibitors, notch signaling pathway, chimeric antigen receptor T-cell therapy (CART) therapy, vascular endothelial growth factor (VEGF), tumor protein p53 (P53 proteins), C-X-C chemokine receptor 4 (CXCR4), and G protein coupled receptor (GPER) receptors targeting [49]. Programmed cell death receptors (PD-1) and check proteins (CTLA-4) prevent the regular mechanism of the body’s defense system from responding to any antigen in case of breast cancer. Targeted inhibition of both PD-1 receptors and checkpoint proteins is employed as an advanced therapy for treating metastatic and advanced BC types [50,51]. Inhibition of miRNA-21 resulted in the anti-proliferation and non-invasiveness of BC cells and the downregulation of oncogenes involved in breast cancer. Therefore, this miRNA-targeted treatment is a suitable choice for treating BC [52].

**Table 1:** Drug-based anticancer targets for breast cancer

Drug	Anticancer mechanism	Type of breast cancer	Cell lines	Inference	References
Tamoxifen	Apoptotic cell death by cell cycle arrest	Not specific	MDA-MB-231 & SKBR-3	Altered gene expression like upregulation of caspase-3 & caspase-9 and downregulation of cyclin D, cyclin E, VEGFR-1, MMP-2 & MMP-9	[64]
Curcumin & Tamoxifen	Apoptotic cell death by cell cycle arrest	All types	MCF-7 & LCC2	By lowering the viability of BC cell lines MCF-7/LCC2 & inducing cell cycle arrest at G phase. Moreover, blocks the hormones responsible for the growth of tumor cells.	[65]
Quercetin	Apoptotic cell death by cell cycle arrest	TNBC	MCF-7 & MDA-MB-231	By inhibiting the tumor metastasis & it also increases the anti-proliferative activity.	[66]
Cabazitaxel & Thymoquinone	Apoptotic cell death	All types of BC	MCF-7 & MDA-MB-231	Apoptotic cell death by targeting multiple pathways	[67]
Posaconazole	Apoptosis	TNBC	MDA-MB-231	The results demonstrated that all assessed features, including cell survival, migratory colony formation, cell cycle analysis, ROS generation, mitochondrial depolarization, and apoptosis, exhibited statistically significant alterations (*p* < 0.01) relative to the untreated control groups.	[68]
Wedelolactone	BC stem cells	TNBC	MDA-MB-231	Prevent drug resistance by downregulation of ABCG2 and SOX2	[69]
SiRNA		TNBC	MDA-MB-231 &BT549	Downregulation of H_3_K_27_ due to ablation of DANCR and altering phosphorylation of various kinases by downregulating the Wnt/EMT signaling pathway	[70]
Baicalin	Epithelial-mesenchymal transition-based metastasis	Metastatic	MCF-7 & SK-BR-3	Directly bound to TGF-β1 caused its activation and regulated the p-Smad3 signaling pathway to make the epithelial cell less mesenchymal	[71]
Resveratrol		All type	MCF-7, MDA-MB-231 & HCC-70	By preventing the aggregation of amyloid, resveratrol also prevents the aggregation of p53C subtype of p53 protein and reduces cell viability and proliferation in breast cancer	[72]
Trastuzumab	Kinase Inhibitor		HER-2	By targeting the overexpressed HER-2 transmembrane receptor to inhibit tyrosine kinase activity. It prevents ligand-independent HER2 signaling	[73]
Alpelisib	PI3K Inhibitor		SKBR3) HER2+ BC cell lines	By blocking the PI3K protein and inhibiting Cell proliferation and differentiation.	[74]
Paris Saponin VII	Ferroptosis	TNBC	MDA-MB-231 and MDA-MB468 and 4TI	By suppression of the proliferation of human BC cells, leading to autophagy and apoptosis	[75]
Olaparib	PARP inhibitor	BC with germline mutations	SUM-149PTHCC-1428 and MDA-MB-231	By inhibiting the PARP enzyme, by prevents DNA repair and induces autophagy	[76]
Doxorubicin	Anti-HER2		HER2-overexpressing BCSKBR-3 cells	Targets the anti-HER2 antibodies	[77]

In the case of people in a state of good health, the process of homeostasis is regulated by the controlled mechanisms of apoptosis and proliferation of cells. This balance is achieved through the presence of certain genes, namely BCLX, BCL-W, and BCL-2, which are located on the mitochondria’s outer membrane and contribute to the cellular count regulation inside the body. However, in the case of breast cancer, anti-apoptotic genes get overexpressed and cause cancer by inducing miRNA degradation and suppressing miRNA transcription [53,54]. Targeted suppression of the BRCA-1 gene signaling pathway and poly (ADP) ribose polymerase (PARP) prevents mutations and non-repairing of breakdown DNA, thereby reducing the chances of cancer progression [55,56]. Tumor proliferation and tumor metabolism are mediated by P13K, an important protein in the phosphoinositide 3-kinase pathway (PI3K)/protein kinase B (AKT)/mammalian target of rapamycin (mTOR) signaling pathway. Therefore, inhibition of the PI3K/AKT/mTOR signaling pathways plays an essential step in a good therapy strategy for breast cancer [57]. Epigenetic modifications cause various cancers; therefore, Histone deacetylase (HDAC) inhibitor, as well as demethylation inhibitors, provides promising and beneficial strategies to overcome TNBC [57]. Inhibition of PARP proteins is an important treatment strategy for BC as it prevents the repair of injured cancerous cells [58]. The inhibition and mutation of MAPK dysregulate the proliferation of residual metastatic cancer cells, specifically BC cells [59]. Furthermore, they are accountable for cell proliferation and growth rate [60]. Notch signaling pathway inhibition prevents mutations in the notch receptors and breaks the association between angiogenesis and the renewal of cancer cells. Hence, it provides anticancer activity and relief from exaggerated BC tumor conditions [61]. Inhibition of Hedgehog (Hh) protein downregulated the embryonic and homeostasis developmental processes in the BC cells [62]. Chimeric antigen receptor (CAR) T-cell (CART) therapy is also known as genetically engineered T-cell therapy. The process in which inactivated T-cells are taken out from the BC patient by apheresis. After that, a chimeric antigen receptor DNA is introduced into the T-cells to activate them, which is then reinserted into the patient’s body. These genetically modified T-cells consider the cancer cells as foreign particles and actively inhibit cancerous cell proliferation [63]. Vascular endothelial growth factors (VEGF) play a crucial role in the multiplication of cellular growth in individuals’ bodies. Moreover, an overly expressed VEGF pathway is critically responsible for the multiplication of cancerous cells. Hence, inhibiting VEG factors like VEGFD, VEGFC, VEGFB, and VEGFA provides effective therapy for BC [61].

## 4 Nano-Size Carrier Systems for Breast Cancer

**Figure 3:**
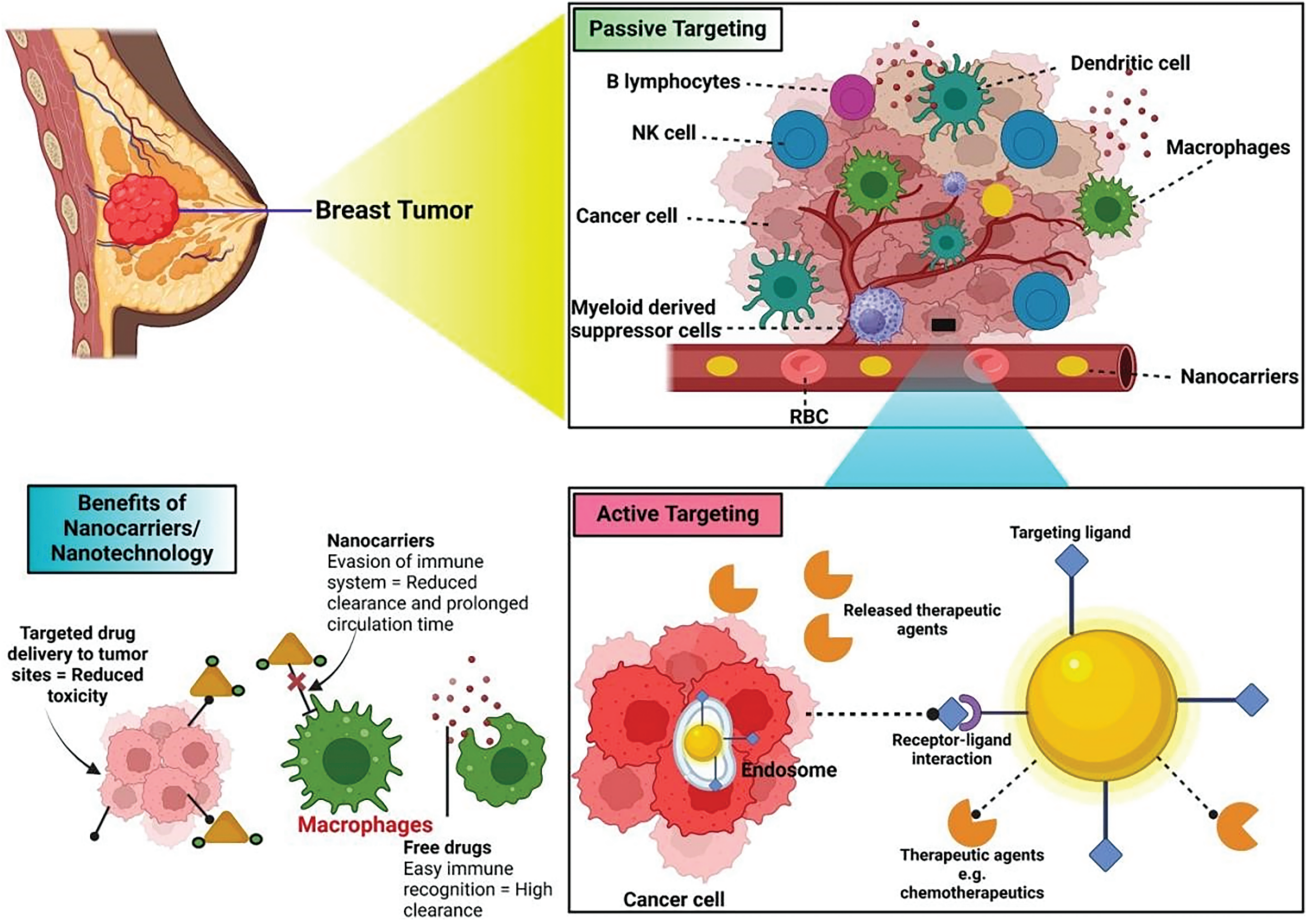
Tumor removal by active and passive targeting in breast cancer

**Figure 4:**
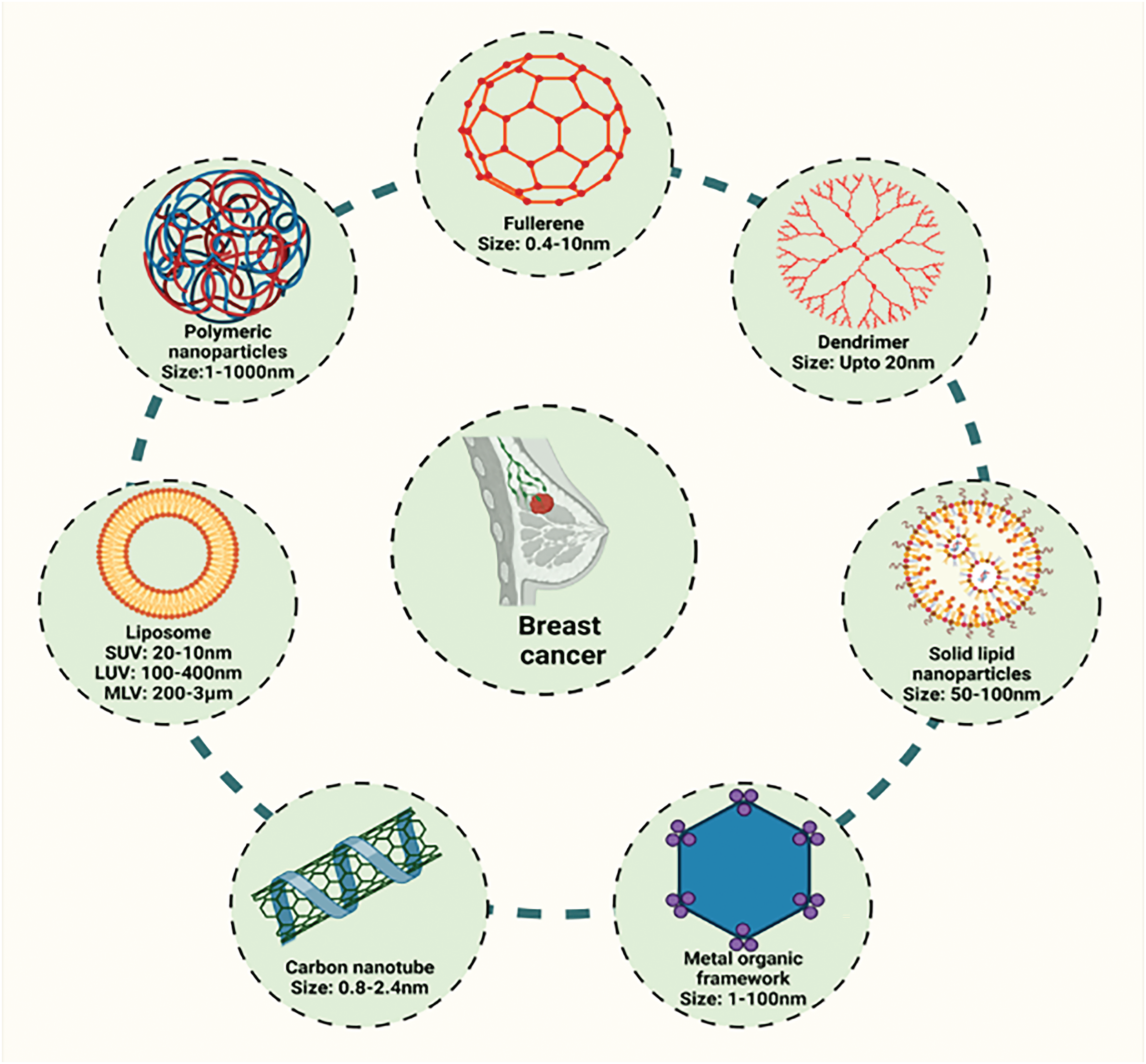
Different nano-based systems for the management of breast cancer

**Figure 5:**
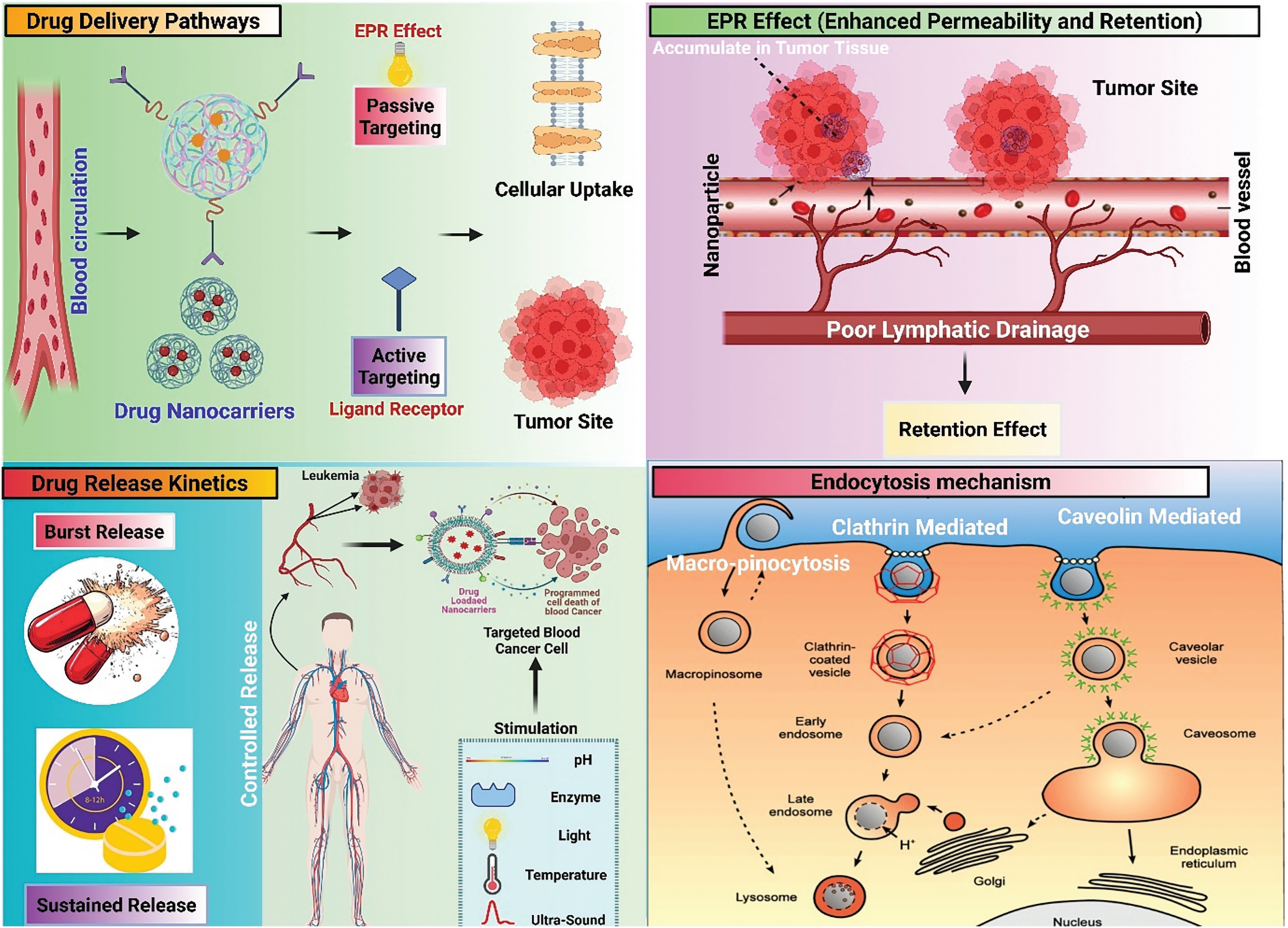
Schematic diagram showing EPR effect, endocytosis mechanisms, and drug release kinetics

Recently from the last decades, nanotechnology has been broadly employed in the area of cancer treatment because of several advantages of nanotechnology, for instance, minuscule size, high capacity of drug loading, sustained drug release, improved stability, efficacy, reduced toxicity due to site-specific drug delivery and increased tolerability of nanocarriers encapsulated with drug particles [78]. Nanocarriers play a crucial role in targeting the tumor microenvironment (TME), which is a complex system influencing cancer progression, immune evasion, and drug resistance (Table 2). TME is a very complicated and heterogeneous environment that consists of cancer cells, stromal cells (e.g., fibroblasts, immune cells, endothelial cells), extracellular matrix (ECM), aberrant vasculature, hypoxia, acidic pH, and high interstitial fluid pressure (IFP) [79]. Thick ECM and abnormal vasculature restrict deep tumor tissue penetration of nanocarriers [80]. Hypoxia and acidic pH may decrease drug efficacy and modify release profiles [81] tumor cells tend to utilize anti-immune mechanisms, which lower the efficiency of immune-based targeting approaches [82]. Smart nanocarriers are designed with surface modification to avoid immune recognition. Camouflaging with cell membranes (e.g., red blood cells, platelets, or tumor cells) offers a natural disguise, evading immune detection [83]. Active targeting ligands help in the functionalization of nanocarriers using antibodies, peptides, aptamers, or small molecules to enhance the selective binding to tumor-associated receptors (e.g., folate receptor, HER2, transferrin receptor) (Fig. 3). pH- or hypoxia-sensitive systems are structured to take advantage of the TME’s specific acidic or hypoxic microenvironment; these systems change their structure or function to facilitate increased cellular uptake [84]. In ECM-cleaving nanocarriers, like Co-delivering hyaluronidase or collagenase assist in breaking down ECM components, allowing greater penetration [85]. In stimulus-responsive release systems, TME-specific triggers (pH, enzymes, reactive oxygen species) trigger smart carriers to deliver the drug exactly at the tumor site, minimizing off-target toxicity [86]. Oxygen-generating nanocarriers help in treating oxygen in hypoxic areas and can improve treatments such as photodynamic therapy (PDT) [87]. Novel drug delivery systems (NDDSs) are inorganic, organic, and hybrid (prepared with two nanomaterials). The design Strategies for integrating nanomaterials with other substances encompass hybrid nanoparticles, the amalgamation of organic and inorganic nanoparticles improves therapeutic effectiveness and stability. Secondly, surface functionalization that modifies nanoparticle surfaces with ligands or antibodies enhances targeted specificity (Fig. 4). Utilizing biodegradable polymers guarantees safe elimination from the body following therapy. Lastly, it includes the biologically controlled release of active materials and should be colloidally stable in physiologically stable [88]. Nanocarriers allow therapeutic drugs to be directly delivered to the cancer cells, which improves treatment outcomes, reduces damage to healthy cells, and combats drug resistance. Nanomaterials surmounted drug resistance by circumventing multidrug resistance mechanisms and enhancing intracellular drug accumulation. Nanoparticles show increased permeability in addition to the retention effect (EPR). The nanocarrier selection for a particulate nano-based system for BC is the most important aspect of the drug delivery systems (Fig. 5), which includes particle size ranging from 10–200 nm, encapsulation of active molecules, and reasonable circulation time. Nanocarriers facilitate the continuous and regulated release of chemotherapeutic drugs, therefore reducing adverse effects [88] (Table 3).

**Table 2:** Information about performance markers

Nano-formulation type	Size (nm)	Polydispersity index (PDI)/Zeta Potential (ZP)	Encapsulation efficiency (%)	IC_**50**_ (**μ**g/mL or **μ**M)	References
Cisplatin nanoliposome	119.7 ± 2.1 nm	−26.03 ± 1.34 mV,	96.65 ± 3%	–	[89]
Piperine-Loaded Polymeric Nanoparticles	107.61 ± 5.28 nm	0.136 ± 0.011, −20.42 ± 1.82 mV	79.53 ± 5.22%	10.12 ± 2.18 μg/mL after 72 h	[90]
Curcumin-loaded solid lipid nanoparticles	310.7 ± 22.3 nm	0.24 ± 0.03 and −26.48 ± 2.35 mV	–	0.85 mg/mL	[91]
Hyaluronic acid NLC containing docetaxel	40 to 247 nm	0.27 and 0.76. 0.26–0.37, 23 mV to −8 mV	–	49.80 nM	[92]
Docetaxel-loaded Niosome	244.9 nm	−7.1 mV	97.43%	–	[93]

**Table 3:** Nano-formulations for breast cancer therapy

Carrier System	Drug	Size range	Cell line/Animal model	Outcomes	References
**Nanoparticles**	Curcumin & HB5 aptamer	281.8 nm	HER2 positive SK-BR3 & HER2 negative	Increased solubility, targeted cellular uptake by HER2-positive SK-BR3 cell lines, hence more cytotoxic in comparison with free curcumin	[94]
**Niosomes**	Curcumin & folate	187.13 nm	4T1 cell lines & MCF-7	BC suppression, targeted drug delivery, suggested it as a promising vehicle for BC therapy	[95]
**Nanoparticles**	Paclitaxel & trastuzumab	180–202 nm	MCF-7 cell lines/rabbits	The medicine exhibited a burst release profile lasting for a duration of 24 h up to 14 days. This release pattern leads to heightened pharmacokinetic characteristics and a decreased rate of clearance	[96]
**Nanoparticle**	Paclitaxel	250 nm	MDA-MB 231 T47D MCF 7 cell lines	Demonstrated reduced tumor volume in treated mice, better antitumor action, no cytotoxicity, and reduced side effects of Paclitaxel	[97]
**Nanoparticle**	Resveratrol	less than 100 nm	MDA-MB231	Induced autophagy in MDA-MB 231 cells, inhibiting invasive BC cells and modulating kinase signaling pathways	[98]
**Nanocapsules**	Folic acid and doxorubicin	1 nm	MDA-MB-231	Formulation had a double surface functionalization, a good targeting ligand, presented high antitumor activity, and reduced cell viability	[99]
**Nanoparticles**	Paclitaxel	105 nm	4T1 tumor-bearing mice	Formulation showed good blood compatibility, inhibited the proliferation of 4T1 cells due to the escalation of therapeutic agent concentration	[100]
**Nanoparticles**	Pazopanib	100 nm	4T1tumor model	It reduced intratumoral infiltration of M2 macrophages. It also suppresses tumor weight and volume. These nanoplatforms act as an effective strategy for combating breast cancer	[101]
**Nanoparticles**	Resveratrol	Less than 5 nm	MGF-7, BALB/c nude mice	Inhibits the NF-signaling system, which provides good biosafety, suppresses the growth of BC, and enhances the good drug delivery in anti-tumor therapy	[102]
**Quantum dots**	Folic Acid Tamoxifen	500 nm	MCF-7 cells	The formulation showed high biocompatibility and pH sensitivity. Antitumor activity. The *in vitro* cytotoxicity studies through the MTT assay confirmed the target and effectively killed BC cells actively	[103]
**Solid lipid nanoparticles**	Paclitaxel	240 nm	MDA-MB231, 4T1 carcinoma cells	Formulation C-peptide-SLN-PTX markedly improved cytotoxicity against 4T1 carcinoma cells. *In vivo*, studies in 4T1 tumor-bearing mice showed an 82% tumor volume reduction, prevented pulmonary metastasis and demonstrated superior antitumor efficacy	[104]
**HA-coated PLGA nanoparticles**	Doxorubicin & indocyanine green	170 nm	(MCF-7) (HTB-22), (MDA-MB231) (HTB-26)	Promising carrier for TNBC with improved efficacy in hypoxic microenvironments. Results demonstrated that CD-44 increased its expression in MDA-MB-231 cells, which can be used for effective targeting and drug delivery in TNBC	[105]
**Solid-lipid nanoparticles**	Doxorubicin & pterostilbene	97.92 nm	MDA-MB-231	These SLNs would improve drug stability, tumor targeting, and therapeutic effectiveness. The results indicated that the best 1:4 (DOX:PTS) ratio had a considerable effect (CI = 0.83). *In vivo*, SLNs enhanced circulation time, tumor accumulation, and decreased tumor volume (701.50 ± 11.83 mm^3^ vs. 3506.58 ± 17.06 mm^3^ control). DOX-PTS SLNs showed that they could work together to fight cancer, stay stable, and distribute drugs to the right place, making them a viable way to treat TNBC	[106]

### 4.1 Dendrimers

Dendrimers have very efficient branching arrangements and are distinguished as three-layered polymeric macromolecules. An asymmetric core nucleus, internal shell, and exterior surface comprise a complicated dendrimer. Dendrimers offer biological and therapeutic applications because of their exact atomic weight, bio-similarity, monodispersed, high solubility in aqueous medium, and high natural border susceptibility (e.g., gene therapy, imaging, and drug delivery) [107]. Dendrimers are considered drug carriers with multifunctional properties since they can change specific drug delivery and regulate release of drugs while improving a dissolution profile, effectiveness, stability, bioavailability, adsorption, and solubility of the drug [45,108]. Medical researchers are researching a variety of dendrimer types as drug carriers, including dendrimers supported by two 2-bis(hydroxymethyl) propanoic acids, and dendrimers based on melamine poly (amidoamine) (PAMAM) dendrimers. According to some reports, dendrimers’ surfaces can be modified to hinder charges and reduce their toxicity. One such modification is PEGylation [109–111]. The findings of the Phase I study indicate that the utilization of a PEGylated PLL dendrimer as a nanoformulation for docetaxel demonstrated enhanced efficacy, safety, and pharmacokinetic properties compared to conventional docetaxel. Similarly, a nanoformulation of SN-38 based on PEGylated PLL dendrimers has progressed to Phase II clinical trials after Phase I trials involving breast, colorectal, and pancreatic cancer patients. These trials demonstrated enhanced anticancer efficacy and improved safety profile in comparison to the conventional irinotecan treatment [112–114]. Zhou et al. developed a polyester dendrimer nanoparticle loaded with hypericin and manganese (MHD) to improve magnetic resonance imaging (MRI) and photodynamic therapy (PDT) using hypericin. They found that the utilization of MHD resulted in a substantial improvement in MRI contrast, owing to the presence of an Mn-based paramagnetic dendrimer carrier. The author also suggested that the suppression of breast tumors through MRI-guided PDT can be accomplished using MHD-carrying hypericin, and the effectiveness of this approach can be further enhanced using Mn^2+^ to increase cellular ROS and alleviate the hypoxic microenvironment [115]. Researchers introduced a new type of dendrimer, called tumor microenvironment (TME)-responsive core-shell tecto dendrimer (CSTD), which demonstrated the effective binding capabilities of CSTD towards Cu^2+^ ions using the anticancer agent disulfiram (DSF) and the further processed complex named CSTD-Cu (II)@DSF. Furthermore, it was shown that the complex has the potential to produce mitochondrial malfunction, demonstrating substantial effectiveness in suppressing the development of MCF-7 tumors [116]. The drugs, namely Fulvestrant and Lapatinib, were utilized in targeted drug delivery of anticancer treatment, specifically for combating BC cells that exhibit distinct phenotypes. Lewińska et al. involved the development of poly (amidoamine) (PAMAM) dendrimer G3, subjected to functionalization with Lapatinib (L) and fulvestrant (F), to evaluate their respective impacts on BC cells. The L-PAMAM and F-PAMAM demonstrated a heightened cytostatic and cytotoxic effect on L and F, respectively, with increased autophagic activity [117].

Chuang et al. created highly adaptable polyamidoamine (PAMAM) dendrimer-based gel nanoparticles that can target both urokinase-type plasminogen activator receptor (uPAR) and ribonucleotide reductase R2 (R2). These nanoparticles were made to attack both TNBC cells and cancer-associated stromal cells by using uPA-uPAR connections and sending the antisense oligonucleotide GTI-2040 (GTI) against R2. The resultant dual-functional dendrimer gel nanoparticles were exceedingly biocompatible. They were around 16.45 nm in size and delivered GTI 3.4 times better in TNBC cells (MDA-MB-231) and 4.8 times better in stromal cells (HCC2218) than GTI alone. It lowered R2 expression by 83.1% and caused about 30% of TNBC cells to die. In a TNBC xenograft model, GDP-uPA/GTI stopped tumor development by 50.5%. These results show that dendrimer gel nanoparticles as promising treatment option for TNBC [118]. Gholamrezazadeh et al. developed and characterized two new nitrophenol-substituted phosphazene dendrimers: [N_3_P_3_(OC_6_H_5_NO_2_)_6_] (III) exhibited reduced fluorescence due to its six 2-nitrophenol substitutions and [N_3_P_3_(OC_6_H_3_(NO_2_)_2_)_5_O]^−^. C_6_H_16_N^+^ · H_2_O (IV) showed elevated fluorescence intensity and toxicity. These findings suggest potential biomedical applications for these cyclophosphamide derivatives, warranting further research into their therapeutic potential [119]. Huang et al. developed a modified generation-5 PAMAM dendrimer to incorporate copper peroxide that which inhibits tumor metastasis, lowers intracellular pH, increases ROS generation, and depletes glutathione (GSH), leading to effective therapeutic outcomes. *In vivo* experiments showed that CuO_2_@G5-BS/TF nano complexes inhibit 4T1 breast tumor growth and metastasis without significant toxicity, offering a novel approach for treating triple-negative BC and enhancing targeted MR imaging [120]. Therefore, rigorous research on rationally crafted dendrimers for breast cancer can open doors to the creation of multiphase dendrimers with outstanding characteristics for combating TNBC in breast cancer therapy.

### 4.2 Lipid-Based Nanocarriers

Nanocarriers based on lipids such as solid lipid nanparticles (SLN), niosomes, and liposomes) have gained considerable attention in drug administration due to simplicity in development, high stability, large-scale manufacture at a cheap cost, biocompatibility, and targetability. Additionally, by providing regulated drug release and increasing drug half-life, they can prolong the effects of drugs. Lipid-based nanocarriers have revolutionized cancer therapy because they have been reported to increase anticancer drug efficacies while lowering toxicities, therapeutic doses, and drug resistance [121,122]. Liposomes are the first developed nanocarriers that are lipid-based for drug delivery. These are sphere-shaped lipid vesicles with at least one phospholipid bilayer surrounding them and an aqueous center. Because phospholipids are amphipathic, they can transport hydrophobic and hydrophilic medications into the phospholipid bilayer and the inner aqueous compartments. PEGylated liposomal doxorubicin was developed in 1995 for treating AIDS-related Kaposi’s sarcoma and was the first FDA-approved nanomedicine. It is now licensed to treat recurrent ovarian cancer, metastatic BC, and multiple myeloma [123–126]. Researchers have designed to deliver site-specific anticancer drug trastuzumab-conjugated Paclitaxel (PTX) and Elacridar (ELA)-loaded PEGylated pH-sensitive liposomes (TPPLs), which resulted in a more significant reduction in tumor burden in the *in vivo* tumor regression investigation with minimal toxicity and hemolysis, along with more drug release percent at an acidic pH = 5 [127]. Ahamad and coworkers designed nanostilbenes that can deliver loaded bioactives to targeted sites and greatly enhance the therapeutic efficiency of encapsulated bioactives, resulting in improved water solubility, decreased breakdown, and enhanced cellular uptake at the site of breast tumors [128]. Lu and his colleague developed a polydopamine (PDA)-coated liposome-based nanoplatform, utilizing both chemotherapy and laser-induced photodynamic and photothermal therapies. This liposomal system consists of Indocyanine green (ICG) and doxorubicin (DOX), resulting in the construction of PDA-liposome nanoparticles, which suggests that it is a versatile nanosystem that utilizes PDA-coated liposomes to facilitate precise and effective combined cancer treatment [129]. This study looks at a new neoadjuvant therapy that uses folate surface-modified liposomes to make an oncolytic Ad with human telomerase reverse transcriptase (Ad-hTERT) work better in CAR-low TNBC tumors. This medication helps lower the level of treatment by lowering or getting rid of the requirement for hazardous chemotherapy combinations or checkpoint inhibitors. *In vitro* investigations using CAR-low TNBC murine 4T1-eGFP cells, CAR-high TNBC human MDA-MB-231-GFP cells, and numerous additional TNBC human cancer cell lines with different levels of CAR expression showed that encapsulated Ad-hTERT was far more hazardous than Ad-hTERT. The same findings were seen in primary TNBC cells taken from patients. *In vivo* studies in immunocompetent mice with CAR-low 4T1-eGFP tumors showed that encapsulated Ad-hTERT, given as neoadjuvant therapy, led to stable or smaller tumors, higher survival rates, more cancer cell death, less cancer cell growth, and more T-cell infiltration in tumors that had been removed. So, liposomal encapsulation of Ad might be a good way to treat TNBC [130]. Maghsoudi et al. synthesized a novel nanocarrier, DOX@m-Lip/PEG, by encapsulating doxorubicin in liposomes containing PEG and Fe_3_O_4_. This nanocarrier was investigated for its potential in treating BC, and it was found that the system is more effective and less harmful to the heart. Moreover, the magnetic characteristic of the m-Lip@PEG nanocarrier renders it a promising material for hyperthermia and MRI investigations [131]. George and his colleague introduce a novel formulation of a newly developed anthraquinone derivative, LipoRV, modified with polyethylene glycol at the nanoscale level. The formulation has demonstrated significant efficacy against the TNBC cell line. The administration of LipoRV effectively impeded the development of the cell cycle, triggered cellular apoptosis, and diminished the extended proliferative capacity of TNBC Cells. In a xenograft animal model, LipoRV successfully cleared tumors and demonstrated a good safety profile, without detrimental effects on biochemical markers. Finally, RNA sequencing of LipoRV-treated TNBC cells was carried out, indicating that LipoRV may have immunomodulatory properties [132]. Li et al. designed a co-loaded crizotinib (Cri) and F7 into a thermosensitive liposome (TSL) to create F7-Cri-TSL. The F7-Cri-TSL was found with high entrapment efficiency (>95%), significant thermosensitive properties, and good stability. On the MCF-7 xenograft mice model, also a therapeutic synergism of Crizotinib, F7, and hyperthermia. Meanwhile, it was shown that the TSL reduced the systemic toxicity of the chemotherapy drug and can function as a viable mechanism for BC therapy that is activated by changes in temperature [133]. Shemesh et al. evaluated a liposomal nano-delivery system in TNBC that used indocyanine green (ICG) as a photosensitizer, which showed that photo-activating liposomal ICG with NIR radiation significantly reduced cell viability by 96% and improved tumor accumulation because of its luminous characteristics. The nano-based liposomal ICG could deliver pharmacological effects and real-time formulation monitoring [134]. Metformin-loaded liposomes, with the help of the Bangham method, were developed. Met-liposomes’ diameter and negative surface charge were 170 and −20 mV, demonstrating reduced TNBC cell proliferation because of the encapsulation of the drug [135]. Li et al. and their colleagues constructed a thermo-sensitive folate receptor-targeted liposome (BI-FA-LP) consisting of Berberine and the photothermal agent Indocyanine green for BC therapy to attenuate the PTT-induced inflammation and inhibit tumor metastasis. BI-FA-LP utilized enhanced permeability and retention (EPR) effect and FA receptor-mediated endocytosis to selectively accumulate at the tumor, reducing off-target toxicity during the treatment. Moreover, BBR could suppress the PTT-induced inflammation, thus inhibiting tumor metastasis and ameliorating tissue injury [136]. This study explores effects of all-trans-retinoic acid (ATRA) and cinnamaldehyde (CA) work together with cisplatin (CPT) in MDA-MB-231 breast cancer cells. Compared to controls, the liposomal formulation CPT_ATRA_CA cut down on cell growth by a lot to 25.9 ± 2.8%, and it also stopped angiogenesis. Also, it caused apoptosis, as shown by flow cytometry, DAPI staining, and a higher Bax/Bcl-2 gene expression ratio. The experimental results, which were backed up by computer data, show that this pharmacological trio has strong anti-tumor effects. These results show that liposomal administration of ATRA, CA, and CPT might improve breast cancer treatment by targeting many pathways at the same time [137]. Various present discoveries have highlighted the role of liposomes as a critical player in the effective treatment of breast cancer. Therefore, a major research effort should go into creating future liposomes that could potentially decrease tumors successfully in breast cancer.

### 4.3 Niosome

Spherical, closed-bilayer structures called niosomes form when non-ionic surfactants, including cholesterol, self-organize into aggregates in aqueous solution [121,138,139]. In contrast, niosomes exhibit enhanced stability compared to liposomes, necessitate less complex manufacturing procedures, and offer a more cost-effective production approach. Niosomes are thus considered a viable alternative for liposomal delivery of anticancer drugs [121]. Sabale et al. developed Capecitabine-loaded niosomes to enhance promising treatment approaches in breast cancer. The optimized batch (F8) exhibited a particle size of 118 nm, a zeta potential of 24.1 mV, an entrapment efficiency of 93%, and a polydispersity index (PDI) of 0.25. The cumulative drug release at a pH of 6.8 indicated that 86.46 ± 0.45% of the drug was released in 24 h. Cytotoxicity testing using MTT assay on MCF-7 breast cancer cell lines showed that the capecitabine niosomes were 2.6 times more cytotoxic than the pure drug. The study demonstrates that capecitabine-niosomes significantly enhanced the anticancer activity of capecitabine, suggesting a promising approach for breast cancer treatment [140].

In this study, a targeted and pH-sensitive niosome (pH-SN) formulation, incorporating quantum dot (QD)-labeled Trastuzumab (Trz) molecules for the specific delivery of Palbociclib (Pal) to cells overexpressing human epidermal growth factor receptor 2 (HER2). Pal encapsulation reached ∼86%, and the release pattern followed a two-phase pH-dependent mechanism. MTT assessments demonstrated enhanced apoptosis induction, particularly in HER2-positive cells, by Trz-Pal-pHSNs. Fluorescence imaging further validated the internalization of particles into cells. In conclusion, Trz-Pal-pHSNs emerge as a promising platform for personalized medicine in the treatment of HER2-positive breast cancer [141]. In the recent investigation by Sharafshadeh et al. (2023), developed niosomes coated with alginate for delivery of cisplatin (Cis) and doxorubicin (Dox), called alginate-coated niosomes (Nio-Cis-Dox-AL), in an attempt to decrease drug doses and overcome multidrug resistance for the effective management of breast cancer. MTT assay showed the IC_50_ of Nio-Cis-Dox-AL was much lower than the Nio-Cis-Dox formulations and free drugs. Cellular and molecular assays demonstrated that Nio-Cis-Dox-AL caused a significant increase in apoptosis induction rate and cell cycle arrest in MCF-7 and A2780 cancer cells and suggested that Nio-Cis-Dox-AL was effectively co-delivered Cis and Dox for the treatment of ovarian and BC [142]. The recent development of niosome nanoparticles as a delivery system for Cyclophosphamide (CYC) and Sodium Oxamate enhances cytotoxicity, induces apoptosis, elevates NRF2 protein levels, and increases expression of caspase-3 and Bax, confirming the activation of apoptotic pathways, leading to effective strategies for managing breast cancer [143]. Jawale et al. developed Plumbagin-encapsulated niosomes to improve the solubility and bioavailability of plumbagin, revealing an extended-release profile. In contrast, niosomes, such as encapsulated plumbagin, are highly effective nanocarriers for enhancing cancer therapy and are promising in drug delivery applications [144]. Moammeri et al. designed niosomal nanocarriers loaded with cisplatin (CIS) and epirubicin (EPI), also consisting of folic acid and polyethylene glycol, to improve endocytosis in the BALB/c mice model. They revealed that the co-administration of CIS and EPI via FA-PEGylated niosomes enhanced the apoptosis rate and cell migration, exhibiting promising prospects for the management of BC [145]. Recent investigational studies reported in the literature have suggested that niosomes can be a potential carrier in the coming years based on their intrinsic characteristics for breast cancer therapy.

### 4.4 Solid Lipid Nanoparticles

A novel class of nanocarriers with enormous therapeutic potential is solid lipid nanoparticles (SLNs). Numerous studies have shown minimal toxicity, good encapsulation, stability, and efficiency. SLNs are submicron-sized colloidal particles with a diameter ranging from 50 to 1000 nm. They comprise a matrix of solid crystalline lipids at room temperature. Antineoplastic drug encapsulation in SLNs has allowed a targeted, regulated, and prolonged release, improving therapeutic efficacy and lowering side effects. Additionally, SLNs’ surfaces can be altered to boost effectiveness [146]. Curcumin Co-Loaded Lawsone SLN with enhanced anticancer efficacy against MCF-7 BC Cell Lines. This SLN was prepared by hot emulsification and probe sonification, whereas TEM studies revealed spherical particles. MTT assay of MCF-7 cells demonstrated increased CUR-LS-SLN cell inhibition and effectiveness, and when combining both drugs with nanocarriers, it produced better inhibition. Moreover, it showed drug synergistic action and sustained drug release for the formulation, and the optimized SLN remained stable for six months as per the stability studies [147]. Chin et al. and their coworkers designed abemaciclib solid lipid nanoparticles to target BC cell lines. The formulation was prepared by Melt emulsification and ultrasonication, followed by Quality-by-Design (QbD) optimization. The study showed enhanced anticancer activity in the MDA-MB-231 and T47D cell lines. In addition, the improved cellular uptake and cytotoxicity, as well as high entrapment efficiency and prolonged release in ABE-SLNs, the application of QbD is a revolutionary strategy that may establish a new benchmark for drug delivery systems based on nanoparticles [148]. Researchers designed atorvastatin and quercetin-loaded solid lipid nanoparticles to combat breast cancer. The studies showed a sustained release profile that performed better than drugs derived from the SLN matrix. The combined action of atorvastatin and quercetin SLNs against MDA-MB-231 was demonstrated in the *in vitro* cytotoxicity studies to be more effective than the two drugs alone. SLNs are a promising drug delivery technology against BC that employs an innovative approach to repurposing well-known drugs [149]. The advent of new SLN technologies for treating breast cancer will provide reliable and selective therapies to patients with BC.

### 4.5 Nanostructured-Lipid Carriers (NLCs)

Nanostructured lipid carriers (NLCs) are an advanced type of lipid-based drug delivery system designed to improve drug stability, bioavailability, and controlled release. They are an evolution of solid lipid nanoparticles (SLNs), incorporating both solid and liquid lipids to enhance drug loading capacity and reduce limitations like drug expulsion during storage.

Passos et al. designed a NLCs for *in situ* thermosensitive drug formulations to prolong drug retention and facilitate ductal administration for BC treatment. Tibutyrin-containing NLCs significantly reduced IC50 values for paclitaxel and 5-fluorouracil in BC cells compared to tricaprylin NLCs. This combination increased the retention of hydrophilic and lipophilic markers in mammary tissue and enhanced cytotoxicity in monolayer and spheroid models, highlighting their potential for improved local BC treatment [150].

Researchers developed a Capecitabine-loaded NLCs to reduce toxicity while treating BC. Results revealed that the niosomes had dramatically increased cytotoxic efficacy, around ten times greater than free capecitabine for a much longer time, as it showed sustained and slow release of capecitabine, effective treatment targeting to tumor site, reducing toxicity, and enhanced therapeutic effects at lower doses, which is therefore considered the preferred cancer treatment [151]. Rodero et al. designed a NLCs loaded with rapamycin with folic acid to overcome drug resistance and the side effects observed also promoted targeting to BC cells, which displayed a particle size of 100 nm, indicating reduced tumor cell viability. *In vitro* studies suggested that FA-NLC-RAP exhibited a higher degree of internalization in cancer cells (MCF-7) than in normal cells (MCF-10A), demonstrating the potential of folic acid as a ligand for promoting active targeting of RAP for breast cancer cells through folate receptors overexpressed in tumor cells FA-NLC-RAP significantly reduced tumor cell viability, similarly to that observed with the RAP solution. The release profile of the formulation was prolonged. Finally, studies in *Caenorhabditis elegans* evidenced the safety of FA-NLC-RAP, characterized by a complete absence of toxicity in this animal model. Therefore, the findings imply that FA-NLC-RAP holds considerable promise for the treatment of breast cancer [152]. Nehal et al. developed a Palbociclib NLCs for BC treatment with reduced adverse effects related to the drug. Intestinal permeation studies demonstrated a 3.76-fold increase in gut permeation with PB-NLC compared to that with PB-Sus. The lipolysis study indicated an enhanced drug availability at the site of absorption. Confocal studies revealed improved drug penetration depth in the intestine with PB-NLC compared to that with PB-Sus. *In vivo* pharmacokinetic studies demonstrated that incorporating PB into a lipidic nanocarrier (PB-NLC) significantly enhanced its bioavailability by approximately 5.9-fold (*p* < 0.05) compared to PB suspension. Additionally, acute toxicity studies in Wistar rats confirmed the safety of the developed NLCs for oral administration in managing breast cancer. Therefore, the PB-loaded NLCs shows significant promise for breast cancer treatment, providing improved drug delivery and minimized side effects [153].

Elkholy et al. repurposed niclosamide for BC treatment but faced issues with its hydrophobicity and low oral bioavailability. They developed NLCs and chitosan-coated NLCs to enhance oral delivery. Furthermore, *in vivo* studies on Ehrlich Carcinoma-bearing mice showed that both NLCs formulations, especially the chitosan-coated NLCs, significantly enhanced niclosamide’s antiproliferative activity compared to the drug suspension [154]. The excellent results on NLCs in the above section demonstrate that NLCs can be satisfactorily manufactured for TNBC as a therapeutic approach.

### 4.6 Polymeric Drug Delivery Systems

Polymer-based nanocarriers provide targeted drug administration and sustained drug release while protecting pharmaceuticals from RES, liver, and renal metabolism and clearance that occurs quickly [155]. They can be made either from natural and/or synthetic polymers. Synthetic polymers are more prevalent than natural ones, have higher mechanical and thermal stability, and may be treated more efficiently to get the necessary scaffold shape and pore size. Natural polymers typically offer higher biocompatibility and biodegradability than synthetic polymers, which can contain impurities that impact their properties [156,157]. He et al. and colleagues utilized a PLGA nanoparticulate platform to encapsulate saturated FA palmitic acid (PA), with DOX or alone, to investigate the potential of PA as a monotherapy or combination therapy for BC and suggest that the PLGA-PA-NPs were as effective in reducing primary tumor growth and metastasis reduced the expression of genes associated with multi-drug resistance and inhibition of apoptosis, and induced apoptosis via a caspase-3-independent pathway in breast cancer cells. In addition, immunohistochemical analysis of residual tumors showed a reduction in M2 macrophage content and infiltration of leukocytes after treatment of PLGA-PA NPs and PLGA-PA-DOX NPs, suggesting immunomodulatory properties of PA in the tumor microenvironment. In conclusion, the use of PA alone or in combination with DOX may represent a promising novel strategy for the treatment of breast cancer [158].

Wang et al. designed a nano-fluorescent polymer drug delivery for BC treatment, where heterometallic coordination polymer (CPI) was synthesized by solvothermal reaction. This novel nanocarrier significantly increased bioavailability and reduced cytotoxicity by exhibiting outstanding fluorescence responsiveness and enabling prolonged drug release in aqueous conditions. The study findings demonstrated that Dimethylamine cations stabilize the compound’s three-dimensional anionic structure, which keeps it thermally stable up to 480°C. Colorless polyimide (CP1) is an excellent option for blue luminescent materials because of its broad blue emission band at 452 nm and exceptional heat stability [159]. Polymeric nanoparticles loaded with Brazilian red propolis were developed by Justino et al. and characterized using Transmission Electron Microscopy (TEM) and Fourier Transform Infrared Spectroscopy (FTIR). While FTIR showed that the extract had been successfully encapsulated, TEM verified the spherical shape and anticipated size of the nanoparticles. Cytotoxicity tests revealed that nanoparticle-encapsulated BRPE (NCBRPE) exhibited reduced toxicity to normal breast cells (MCF-10) but increased toxicity to breast cancer cells (MCF-7) compared to free BRPE, suggesting targeted therapeutic potential. Notably, NCBRPE demonstrated enhanced efficacy in acidic tumor microenvironments, further supporting its targeted therapeutic potential for breast cancer therapy [160]. Chaudhari et al. developed poly (lactic-co-glycolic acid) (PLGA) loaded with paclitaxel (PTX) and conjugated with adenosine (ADN) to target triple-negative BC (TNBC), in which the ADN component of the nanoparticles serves as a substrate for adenosine receptors (AR), which are known to lie on the surface of TNBC. The study’s findings indicate that the optimized particles exhibited biocompatibility and enhanced anti-TNBC activity [161]. PLGA nanoparticles co-loaded with the anticancer agents, namely curcumin and paclitaxel, and the system referred to as PLGA-CUR-PTX nanoparticles. The result of this investigation suggested that the optimized nanoparticles exhibited a superior cytotoxic effect, as evidenced by reduced IC50 values, against the MCF-7 and 4T1 BC cell lines compared to the unbound drugs [162]. Sarpoli et al. synthesized a novel nanostructure PLGA-polyethyleneimine (PEI) nanosystem with the purpose of co-delivering curcumin and siRNA to BC cells and suggested that the nanosystem exhibited notable cytotoxic effects on the T47D cell line by delivering an anticancer drug for the effective management of BC cells [163]. Hu et al. and their team synthesized hyaluronic acid-coated Olaparib-loaded PEI-PLGA nanoparticles. They evaluated their efficiency as a site-effective anticancer agent, and sustained drug release behavior exhibited efficient *in vitro* and *in vivo* antitumor activities. HA-Ola-PPNPs induced cell apoptosis by upregulating Bax, Cytochrome C, and Caspase 3, downregulating Bcl-2 in breast cancer-bearing mice against tumors and suggested that nanoparticles loaded with Olaparib potently target triple-negative BC cells [164].

Malarz et al. developed pH-sensitive phthalocyanine-loaded polymeric nanoparticles as a novel treatment strategy for combating BC. However, Polymeric nanoparticles coated with pH-sensitive phthalocyanines selectively inhibit the growth of BC cells while leaving healthy cells unaltered. Photocytotoxicity experiments demonstrated their excellent efficacy and selectivity for the SK-BR-3 cell line, confirming their high antiproliferative activity [165].

Behl and his research team created a novel nanoformulation, called DM-PEG-PCL NPs, comprising a polyethylene glycol-polycaprolactone (PEG-PCL) polymer that is packed with a MUC1 inhibitor and a potent anticancer medication, doxorubicin (DOX), for the treatment of TNBC. The delivery technology under control exhibits biodegradability, non-toxicity, and anti-multidrug-resistant properties. Furthermore, the customized smart nanoformulation has demonstrated notable efficacy in treating TNBC [166].

Liaqat et al. synthesized zinc selenide quantum dots (ZnSe QDs) and combined them with a doxorubicin sitagliptin-lignin biopolymer (SL) for a drug delivery system. Optimal drug release occurred at pH 6.5 and 45°C, achieving 81.75% drug encapsulation. SLQD-Doxo demonstrated 24.4% anti-inflammatory activity and 71.45% lipoxygenase inhibition. Tested on MCF-7 BC cells, it resulted in 74.39% cell viability and 24.48% cell death, significant therapeutic, anti-inflammatory, antioxidant, cytotoxic, and increased antioxidant activity. The lignin’s polyphenolic nature contributed to vigorous antioxidant activity, making SLQD-Doxo effective for tumor cells’ high temperatures and acidic pH [167].

Odeniyi et al. designed thiolated and carboxymethylated Jackfruit seed starch nanocrystals as polymeric drug carriers for the delivery of curcumin to cancer Cells. This study assessed modified jackfruit starch nanocrystals as potential delivery systems for curcumin to MCF-7 BC cell lines. The cytotoxicity and *in vitro* drug release of curcumin-loaded starches on MCF-7 cancer cells were assessed. The produced nanocrystals showed a modified morphology and an average particle size of 6.15 ± 0.80 nm. Carboxymethylated nanocrystals had the highest drug loading (70.23% ± 0.23%), followed by thiolated nanocrystals (63.37% ± 0.40%), while native starch had the lowest (49.31% ± 0.13%). Therefore, modified jackfruit starch nanocrystals are acceptable alternatives for the efficient delivery of curcumin to cancer cells because they have desirable qualities [168].

Nasr et al. developed folic acid (FA) grafted polymeric micelles loaded with tamoxifen citrate (TMXC) to enhance antitumor activity in breast tissues. *In vitro* evaluation showed that the FA-conjugated micelles (FA-P123/P84) had an encapsulation efficiency of 87.83%, a size of 35.01 nm, and a surface charge of −20.50 mV. These micelles released 67.58% of TMXC after 36 h and demonstrated 2.48 times better cytotoxicity and higher cellular uptake in MCF-7 cells. *In vivo* studies showed significant tumor volume reduction in tumor-bearing mice treated with TMXC-loaded FA-P123/P84, suggesting these micelles are a promising carrier for TMXC in BC treatment [169].

Sah and Kumar designed PLGA-SPC3 functionalized gefitinib mesoporous silica nano-scaffolds for BC by using a heat-assisted method. In optimized MSN, albino Wistar rats were used for the *in vivo* biodistribution investigations, cytotoxicity measurement, and *in vitro* drug release kinetics. The outcome indicates that MSN’s surface area, pore volume, % encapsulation efficiency (EE), and drug loading increased. Moreover, *in vitro* cytotoxicity showed more anticancer activity than free drug (43.84% ± 0.63%, *p* < 0.05. However, its effectiveness in treating BC was demonstrated by the rise in cell viability observed in the MCF-7 cell line during *in vitro* tests; therefore, a novel approach for combating BC [170].

Lv et al. designed folate-functionalized carboxymethyl chitosan (CMCS)/calcium phosphate hybrid nanoparticles (CF/CaP) with Ca^2+^ production to treat BC by combining them with encapsulated curcumin (Cur). The spherical C@CF/CaP nanoparticles, about 179 nm, showed strong stability, acid-responsive drug release, and good biocompatibility. At pH 5.0, over 70% of Cur was released after 36 h. These nanoparticles targeted tumor tissues via folate receptor-mediated endocytosis, causing mitochondrial Ca^2+^ overload, initiating the mitochondrial apoptotic pathway, and damaging the mitochondrial structure. The study demonstrated that CMCS-based Ca^2+^ nano-modulators could be a viable organelle-targeting approach for cancer treatment [171]. Rakhshani et al. designed doxorubicin PLGA nanoparticles and simvastatin loaded in silk films (SV/DOX PLGA/SF films) for local treatment of BC. The study revealed uniform size and spherical morphology. In female Balb/c mice, SV/DOX PLGA/SF films showed the most significant tumor suppression effect and the lowest tumor recurrence, with only around 52% ± 10.3% of the original tumor remaining. Furthermore, SF-based localized therapies dramatically decreased the mitotic index, according to histological investigation using H&E staining. Therefore, it is biocompatible, biodegradable, and used for post-surgical cancer combination treatment [172].

### 4.7 Protein-Based Drug Delivery Systems

Multiple protein subunits make protein-based nanocarriers, which can self-assemble precisely and spontaneously to create hollow cavities with internal nanocarriers [173]. Due to the unique characteristics of protein-based nanocarriers, their practical applications (such as biocatalysis, diagnostic imaging, drug administration, and vaccine development) have rapidly increased during the past few years [174]. In addition to their biocompatibility and biodegradability, protein-based nanocarriers are also easy to synthesize, control size, are inexpensive, have strong stability, can be modified on the surface for targeted drug delivery, and can be used to control drug release. Nevertheless, the utilization of nanocarriers derived from diverse proteins is not without its limitations. These drawbacks encompass the considerable expense associated with specific proteins like albumin and ferritin, the possible risk of prion transmission from animal sources, like collagen and gelatin, the poor mechanical integrity of gelatin, and the slow or fast degradation of silk protein fibroin [175,176]. The field of oncology has demonstrated the most significant utilization of protein-based nanocarriers.

Wang et al. developed cinchonine and immunoglobulin G nanoparticles in order to improve anti-BC activity. This study revealed that the produced Cin-IgG NPs exhibit a pH-sensitive drug release behavior, a hydrodynamic size of 190 nm, and an EE% of 72.38%. Furthermore, Cin-IgG NPs have a more substantial effect on the downregulation of PI3K/p-AKT and the overexpression of the Bax/Bcl-2 ratio compared to the free drug. This study concludes that Cin can bind IgG as a human plasma protein and that complexing with IgG to generate an NP form can enhance its anti-BC properties [177]. Deng et al. developed silk protein-based nanoporous microspheres using an Ionic Liquids (ILs)-induced self-assembly process blended with poly (d,l-lactic acid). These microspheres exhibited effective drug loading (up to 88.7%) and sustained release kinetics, with over 53.5% drug release in the first four hours. The ILs facilitated hydrophobic and electrostatic interactions, enabling drug entry into the nanoporous matrix and inducing reversible changes in protein structure during delivery. The system shows promise as an efficient drug delivery vehicle for various biomaterial applications [178].

Are and her coworkers designed nanoparticles using human serum albumin (HSA), which was coupled with vitamin E and loaded with a tyrosine kinase inhibitor (TKI) or an aromatase inhibitor (AI) with the help of the desolvation method. Some physicochemical tests, such as Infrared spectroscopy (IR), gel permeation chromatography (GPC), ultraviolet, infrared, and CD spectroscopy, were used to study HSA (VE) binding and drug incorporation into nanoparticles, assessing their drug entrapment and release efficiency. Eventually, cell viability tests and *in vitro* studies on resistant and sensitive cell lines. The study showed efficient drug encapsulation and absorption. Studies conducted *in vitro* and *in vivo* showed that a 75:2 lapatinib-loaded nib-loaded HAS-VE NPs to Letrozole loaded HAS-VE NPs reduced tumor growth and increased apoptosis compared to individual NP therapy and free drug. In addition, when VE and HSA were conjugated, the outcome was self-assembly into nanoaggregates with enhanced cellular absorption, decreased IC50 values, and a high drug loading capacity [179].

Safwat et al. developed bioinspired caffeic acid-laden milk protein-based nanoparticles targeting folate receptors for BC treatment prepared using a simple coacervation method and lyophilization. This study demonstrated that conjugated NPs achieved IC_50_ = 40 ± 2.9 μg/mL, and PS = 157.23 ± 2.64 nm. In the tumor-induced animals treated with conjugated NPs, there was a significant decrease in the biochemical marker levels of malondialdehyde, carbohydrate antigen 15−3, and carcinoembryonic antigen, and an increase in superoxide dismutase. Histopathological analyses revealed significant improvement in the necrotic and mammary areas. Moreover, these findings highlight the most promising platform for showcasing advanced therapeutic applications for combating various diseases, including breast cancer [180]. As research continues to develop, these novel approaches, like protein-drug delivery, show a promising arena in the creation of customized, next-generation cancer treatment.

### 4.8 Carbon-Based Nanocarriers

Carbon-based nanocarriers have also been made because of their features, notably high chemical stability, large surface area, preferential tumor accumulation, and high cellular penetration. They are now potentially promising medication carriers for cancer treatment [181,182]. Bovine serum albumin (BSA) and folic acid-coated platinum-functionalized oxygenated single-walled carbon nanotubes (O-SWCNTs-Pt to create O-SWCNTs-Pt-BSA-FA. The resulting nanotubes were then utilized as radiosensitizers to enhance the therapeutic effectiveness of X-rays in an *in vitro* mouse model of BC (4T1). The novel nano-sensitizer can be utilized with radiotherapy technology for cancer treatment, as it showed very strong cell-killing activity in the 4T1 cell line, controlled release, and easy penetration into biological membranes [183]. Badea et al. investigated the fabrication of a nanoconjugate utilizing single-walled carbon nanotubes with single-walled that were functionalized with cisplatin (CDDP) and carboxyl groups (SWCNT-COOH-CDDP) were compared them with conventional 2D- and 3D-spheroid cultures of human breast cancer cells. The SWCNT–COOH–CDDP complex proved to have high anti-cancer efficiency on 2D and 3D cultures by inhibiting cell proliferation and activating cell death. A dose of 0.632 μg/mL complex triggered different pathways of apoptosis in 2D and 3D cultures, by intrinsic, extrinsic, and endoplasmic reticulum pathways. Overall, the 2D cultures showed higher susceptibility to the action of complex compared to 3D cultures, and SWCNT–COOH–CDDP proved enhanced anti-tumoral activity compared to free CDDP [184].

Xie et al. developed a carbon nanoparticle with Fe (II) complex (CNSI-Fe) combined with sorafenib for ferroptosis-induced (SRF) antitumor effects in triple-negative breast cancer. In this study, CNSI-Fe, in conjunction with sorafenib (SRF), a ferroptosis inducer, can provide adequate treatment for TNBC. With a maximal adsorption capacity of 31 mg/g, CNSI-Fe could adsorb SRF through hydrophobic contact and π-π stacking. Compared to CNSI-Fe or SRF alone, the CNSI-Fe+SRF combination significantly reduced the cell viability of 4T1 cells during the *in vitro* tests. The oxidative damage, hydroxyl radical production, and elevated Fe absorption confirmed that 4T1 cells underwent ferroptosis following CNSI-Fe+SRF therapy. SRF improved the therapeutic impact of CNSI-Fe during the *in vivo* tests, as evidenced by the greater survival rate and tumor growth suppression rate of 67.8%. Therefore, it is a promising and efficient treatment for TNBC through ferroptosis [185].

Graphene oxide nanoparticles (HA-GO-Met) loaded with metformin grafted with hyaluronic acid (HA) showed antitumor effectiveness at far lower doses than metformin alone, according to a 2021 study. Cell migration was inhibited by the reduction of pFAK/integrinβ1 expression. Treatment inhibited epithelial-mesenchymal transition (EMT) and reduced stemness as evidenced by the increase in E-cadherin expression, inhibition of mammosphere formation, and low expression levels of stemness markers, including nanog, oct4, and sox2, as compared to control. Moreover, tumor regression was studied in chick embryo xenograft and BALB/c mice models. HA-GO-Met nanoparticle treatment reduced tumor load and nullified toxicity in peripheral organs imparted by the tumor [186]. Carbon dots doped with gadolinium, Fe^3+^, and Mn^2+^ have been demonstrated to have been developed from N-hydroxyphthalimide. Cell viability was assessed after doped carbon dots were applied to normal and cancerous cell lines. It was found that Mn^2+^ doped Carbon Dots (Mn-CDs-NHF) had antitumor properties without compromising the viability of normal cells and decreased the size of primary mammary tumors while permitting magnetic resonance imaging, implying that they can be employed in preclinical models as theranostic agents [187]. Radzi et al. prepared an oxidized multi-walled carbon nanotube (ox-MWCNTs) developed by acid washing and hypothermic breast cancer treatment. The prepared formulation was found to eliminate the tumor when tested *in vivo* in mice. Histopathology analysis revealed that tumors treated with the intended treatment underwent necrosis. Also, there was a significant increase in natural killer cells, macrophages, and CD8+ and CD84^+^ T cells in the tumor cells. The study’s overall findings point to the potential of ox-MWCNTs-mediated HT as an anticancer therapeutic agent, which may prove advantageous for the treatment of BC in the future [188].

Tiwari et al. developed a method to reduce chemotherapy toxicity by encapsulating dacarbazine (DC) in fucose-based carbon quantum dots (CQDs) and then coating them with exosomes from BC cells (Ex-DC@CQDs). Western blotting and nanoparticle tracking analysis confirmed that Ex-DC@CQDs retained exosome properties. Exosomes enhanced delivery to cancer cells via HSPG receptors, increasing mitochondrial membrane potential depolarization, ROS production, and apoptosis. *In vivo*, Ex-DC@CQDs showed more significant tumor accumulation due to sustained release and exosome-mediated uptake. This approach suggested biologically sourced nanocarriers as a promising future for BC treatment [189]. Varvarà et al. designed targeted NIR-triggered doxorubicin release using carbon dots–poly(ethylene glycol)–folate conjugates for effective treatment. Doxorubicin-loaded CDs (CDs-bAPAE-PEG-FA/Dox) exhibited on-demand NIR-boosted drug release, which is increased by 50% after localized NIR exposure, whereas *in vitro* studies on BC cells MCF-7 and MDA-MB-231 exhibited NIR-enhanced antitumor efficacy, which reveals selective and remote-controlled synergistic therapy. According to data, CDs-bAPAE-PEG-FA/Dox could carry out effective image-guided and selective BC therapy, improving the therapeutic results [190].

Carbon nanocarriers, including graphene, carbon nanotubes, and fullerenes, hold promise for drug delivery but also present challenges in terms of off-target toxicity, biodistribution, and immune response. Carbon nanocarriers may accumulate in non-target organs, resulting in unwanted cytotoxicity. Certain carbon-based materials cause oxidative stress, which hurts normal cells. Nonhomogeneous distribution of nanoparticles may lead to suboptimal delivery of drugs, decreasing therapeutic efficacy. Some nanocarriers show extended retention in the spleen and liver, impacting clearance. Carbon-based nanocarriers can interact with immune cells, and this may result in immunosuppression or overstimulation. Surface modifications are under investigation to prevent immune system activation and enhance biocompatibility.

### 4.9 Nanocomposites

Nanocomposites are a subset of deliberately engineered materials known as nanomaterials, which integrate nanoscale particles inside a matrix of conventional materials. Nanosized particles may possess one, two, or three dimensions measuring less than 100 nm. The incorporation of nanoscale components confers distinctive properties to the composite material, rendering nanocomposites advantageous for many applications, including healthcare.

Silica-based nanocomposites are used for biosensing and targeted therapy due to their porous structure. Nanomaterials interact with cell membranes, facilitating targeted drug delivery [191].

This investigation used green synthesis of a bi-metallic nanocomposite of nickel and zinc using an aqueous extract of *Citrus sinensis* in the presence of chitosan (Ni/Zn@orange/chitosan). The NPs were spherical in shape and size of 17.34–90.51 nm., The composite nanoparticles were studied by the MTT method for the anti-breast adenocarcinoma potential in malignant cell lines. The results revealed that the newly synthesized nanocomposite is a potent photocatalyst and an acceptable new drug to treat breast cancer [192].

### 4.10 Hybrid Nanomedicine

Hybrid nanomedicine means combination therapies that include chemotherapeutics, and multiple chemotherapeutics, along with biologicals, polydynamic, and chemotherapeutics, radiation, and chemotherapeutics. It contains both inorganic and organic components. The potential to combine with mass organic and inorganic components to achieve the desired hybrid nano-formulation that allows the systemic alteration to achieve the desired results. Hybrid nanomaterials and gold-coated iron oxide nanoparticles are used for imaging and drug delivery. Hybrid nanogels integrate organic and inorganic components for controlled drug release [193].

ISL-loaded zein phosphatidylcholine hybrid nanoparticles (ISL@ZLH NPs) were constructed by a one-step solvent evaporation method. Optimization and characterization of ISL@ZLH NPs were performed. Cellular uptake *in vitro* and biodistribution of ZLH NPs *in vivo* were investigated. The pharmacokinetics of ISL@ZLH NPs in terms of ISL content in the plasma, organs, and tumor tissues were validated, and the anti-TNBC efficacy was evaluated. Encapsulation efficiency (96.75 ± 1.41%) and drug loading efficiency (6.56 ± 0.83%) were found in ISL@ZLH NPs. ISL@ZLH NPs were able to enhance the absorption of ISL in the tumor sites. Moreover, the upregulation of p27 and downregulation of EGFR and CDK4 were observed in ISL@ZLH NPs-treated tumors. Collectively, oral intake of ISL@ZLH NPs would be translated into a potential clinical therapy strategy against TNBC [194].

Designing multiphase nanomaterials can be done by doping with metals like silver or gold to enhance antibacterial and photocatalytic properties by doping with nitrogen or boron to improve conductivity and biocompatibility. Combining polymers with metal oxides for improved drug delivery and stability.

### 4.11 Nanotoxicity and Associated Risks

Numerous nanocarriers encounter difficulties in accurately targeting breast cancer cells, resulting in off-target consequences and diminished therapeutic efficiency. Certain nanoparticles are unable to surmount multidrug resistance processes, hence constraining their efficacy in prolonged therapy. The synthesis and scalability of nanocarriers present significant challenges, obstructing their extensive use. Some nanomaterials demonstrate cytotoxicity, prompting questions over their safety for therapeutic use. Many inorganic NDDSs exhibit cytotoxicity, limiting their clinical applications. Organic NDDSs often suffer from rapid degradation, reducing drug efficacy. Hybrid NDDSs sometimes fail to achieve optimal targeting due to inconsistent surface modifications. The synthesis of NDDSs requires precise control over size, shape, and composition, making large-scale production challenging. The long-term effects of NDDSs on human health and the environment remain inadequately studied. The interaction between NDDSs and biological systems is not fully understood, affecting their predictability in drug delivery. There is a lack of standardized protocols for evaluating NDDS efficacy and safety. Despite promising preclinical results, few NDDSs have successfully transitioned to clinical applications.

Nanotoxicology, an essential component of nanoscience, examines the possible detrimental consequences of manufactured nanoparticles with diverse mechanisms, particularly in biological applications. Nanoparticles (NPs) are extensively utilized in nanomedicine; yet, their nanosize and unique properties raise concerns over their safety, along with some other factors like size, shape, surface functional groups, and dose-dependent characteristics that also influence toxicity. Chemically synthesized nanoparticles may have elevated toxicity due to synthetic compounds, whereas biosynthesized nanoparticles with biocompatible surface groups indicate reduced toxicity [195]. About human health, dose-dependent nanoparticle toxicity refers to the escalation of a particle’s harmful effects with higher concentrations or extended exposure. At lower quantities, they may be benign or even beneficial; nevertheless, at elevated concentrations, they can induce oxidative stress, inflammation, and DNA damage, and even result in cellular death. The toxicity of a nanoparticle is contingent upon its method of exposure (ingestion, inhalation, etc.) and the degradation of nanoparticles into simpler forms or their accumulation within cells. Prolonged or elevated exposure heightens the risk of cancer and adversely affects the respiratory, cardiovascular, and neurological systems [196,197]. Nanotoxicology aims to detect possible risks and evaluate the safety of nanomedicines. The subsequent section reviewed contemporary research that offered recent insights into the toxicological consequences linked to nanoparticles and the principal processes driving nanoparticle toxicity. Specifically, concentrating on the potential of nanoparticles for direct contact with the cell membrane, their capacity to dissolve and release harmful ions, and their propensity to trigger oxidative stress. Research indicates that nanoparticles (NPs) can infiltrate the human body by inhalation, ingestion, and topical application, potentially causing harm to adjacent cells, tissues, and organs. Recent studies have emphasized the exploration of the processes behind the toxicity of nanomaterials, particularly their adhesion to the cell membrane. The interaction of nanoparticles with cells or cellular components, including the cell membrane, metabolic processes, ribosomes, mitochondrial activity, and the electron transport chain, influences their toxicity [198].

The morphology of nanoparticles influences their interaction with cellular membranes, behavior in the circulatory system, and their dispersion throughout various tissues. For example, rod-shaped or needle-like nanoparticles may infiltrate cell membranes more readily than spherical nanoparticles, potentially resulting in more damage and heightened toxicity. Various geometries can affect the mechanism and efficacy of cellular absorption. Shapes that are more easily absorbed may result in elevated intracellular concentrations of nanoparticles, hence increasing toxicity [199]. Non-spherical nanoparticles may exhibit modified clearance rates and tissue penetration, possibly resulting in a buildup in certain organs and heightened local toxicity [200]. Various nanoparticle morphologies may obstruct their elimination from the organism, causing extended circulation durations, resulting in bioaccumulation and possible chronic health repercussions [201]. Shape management is essential, as various shapes interact with cell membranes and tissues distinctly, influencing cellular uptake, membrane integrity, and biodistribution, all of which contribute to nanotoxicity.

To ensure the safe use of nanoparticles, researchers are developing strategies such as coating nanoparticles with biocompatible materials, controlling nanoparticle size to improve biodistribution and minimize unintended effects, and establishing safety protocols for nanoparticle exposure in medical and industrial applications. Nanotechnology offers immense potential, but understanding its risks is essential for responsible innovation.

## 5 Clinical Trials, Ethical and Regulatory Considerations in Nanomedicine for Breast Cancer

Clinical trials are human research studies carried out by different commercial organizations throughout the globe to develop new treatments with more potential and less toxicity. Various clinical trials of drug moieties, biological compounds, and their combinations are running for the BC treatment. For BC therapy, clinical trials of different stages and FDA-approved drugs are listed below (Tables 4 and 5). The patented technology is also discussed in tabular form (Table 6). Nanomedicine is transforming breast cancer therapy by providing precision-targeted medicines and improved drug delivery systems. Before these improvements can be used in clinical practice, they must traverse a multifaceted terrain of ethical concerns, clinical trial mandates, and regulatory approvals. Researchers and physicians must confront the following ethical issues: patient safety and risk mitigation, due to the potential accumulation of nanoparticles in unexpected organs, and their long-term consequences are indeterminate. Ethical principles emphasize thorough preclinical safety evaluations to reduce health hazards [202].

**Table 4:** Clinical trials conducted for breast cancer

Status	Trial no.	Sponsor	References
**Recruiting**	NCT03572361	Immunitor LLC.	[206]
**Recruiting**	NCT04072952	Arvinas Estrogen Receptor, Inc.	[207]
**Recruiting**	NCT05104866	AstraZeneca	[208]
**Recruiting**	NCT01969643	Seagen Inc.	[209]
**Active, not recruiting**	NCT04579380	Seagen Inc.	[210]
**Phase 2, recruiting**	NCT01042379	QuantumLeap Healthcare Collaborative	[211]
**Active, not recruiting**	NCT04539938	Seagen Inc.	[212]
**Active, not recruiting**	NCT04434040	Dana-Farber Cancer Institute	[213]
**Recruiting**	NCT03971409	Hope Rugo, MD	[214]
**Completed**	NCT01644890	Nippon Kayaku Co., Ltd.	[215]
**Recruiting**	NCT03671044	Jina Pharmaceuticals Inc.	[216]
**Recruiting**	NCT04137653	Shengjing Hospital	[217]
**Recruiting**	NCT04917900	West China Hospital	[218]
**Recruiting**	NCT06143553	Shanghai Yizhong Pharmaceutical Co., Ltd.	[219]
**Recruiting**	NCT06199895	Liu Huang	[220]
**Recruiting**	NCT05949021	Mridula George, MD	[221]
**Recruiting**	NCT05781633	Tianjin Medical University Cancer Institute and Hospital	[222]
**Ongoing (phase 3)**	NCT04483007	National Cancer Institute	[223]

**Table 5:** FDA-approved drugs for breast cancer

Drug Name	Phase status	Number of participants	Indications	Sponsor	References
Inavolisib + Fulvestrant + palbociclib	Approved	325	PIK3CA-mutated, HR+/HER2+ metastatic breast cancer	Genentech, Inc.	[224]
Elacestrant	Approved	478	ESR1 mutated, ER+, HER2- metastatic breast cancer	Stemline Therapeutics, Inc.	[225]
Fam-trastuzumab- deruxtecan-nxki	Approved	866	HER-2 low breast cancer	Daiichi Sankyo, Inc.	[226]
Datopotamab-deruxtecan-dlnk	Approved	732	HR+, HER2- breast cancer	Daiichi Sankyo, Inc.	[227]
Capivasertib+ Fulvestrant	Approved	708	HR+, HER2- metastatic breast cancer	AstraZeneca Pharmaceuticals	[228]
Abemaciclib	Phase 3	5637	HR+, HER2-, node-positive early breast cancer	Eli Lilly and Company	[229]
Alpelisib + Fulvestrant	Approved	571	HR+, HER2- PIK3CA mutated, advanced breast cancer	Norvartis Pharmaceuticals Corporation	[230]
Sacituzumab-govitecan-hziy	Approved	543	HR+, HER2- breast cancer	Gilead Sciences, Inc	[231]
Ribociclib + letrozole	Approved	5101	HR+, HER2- stage 1 and 2 early breast cancer	Norvartis Pharmaceuticals Corporation	[232]

**Table 6:** Patents in breast cancer

Patent no.	Title	Highlights	Date and references
**US20240360068A1**	Curcumin analogues as zinc chelators and their uses	Prior research has demonstrated that curcumin decreases the MMP-2 expression in BC cell lines, and highly metastatic cells become less aggressive. Furthermore, it decrease in MMP-2 activity may be a key factor in curcumin’s anti-metastatic effects.	31 October 2024 [233]
**AU2024259755A1**	Viral delivery of neoantigen	Neoantigen-based vaccination can elicit T-cell responses, resulting in neoantigen-targeted cell therapy that can cause tumor regression.	21 November 2024 [234]
**AU2024259800A1**	Systems, methods, and compositions for targeted nucleic acid editing	It is used in BC to prevent familial 1 breast-ovarian cancer by correcting the mutation or SNP.	28 November 2024 [235]
**AU2024227645A1**	T cell receptor constructs and uses thereof	T cells expressing TCRs against peptide-MHC complexes are used in optimal usage in the treatment of breast cancer, TNBC, etc.	14 November 2024 [236]
**AU2024227446A1**	ROR1 antibody immunoconjugates	Immunoconjugates comprise an anti-RORI antibody and a drug moiety. These immunoconjugates are useful for treating RORI-expressing cancers. Moreover, they are used to correct the pathogenic mutations or SNPs associated with familial 1 breast-ovarian cancer.	07 November 2024 [237]

Nanotherapeutics sometimes entail substantial research and production expenses, prompting apprehensions over affordability and accessibility for disadvantaged patients. The equitable allocation of sophisticated cancer therapies is an increasingly significant ethical concern. Nonetheless, its clinical application needs rigorous regulatory supervision to guarantee safety, effectiveness, and ethical adherence [203]. The Food and Drug Administration (FDA), European Medicines Agency (EMA), and International Council for Harmonisation of Technical Requirements for Pharmaceuticals for Human Use (ICH) are pivotal in formulating criteria for the approval of nanomedicine. The FDA offers explicit recommendations for medicinal products utilizing nanotechnology, emphasizing manufacturing processes, pharmacokinetics, and toxicity evaluations. Guidelines for assessing nanoparticle interactions with biological systems to mitigate toxicity concerns. Focus on extensive biodistribution studies and assessments of immune responses for nanomedicine-based therapies. The EMA has created horizon-scanning reports to evaluate developing nanomedicines, focusing on categorization, regulatory readiness, and ethical issues. Essential factors encompass the continuous assessment of nanoparticle stability, toxicity, and therapeutic effectiveness. Assessing the effects of nanomedicines on ecosystems and human health. Protocols for connecting preclinical discoveries with human trials to improve regulatory approval procedures [204]. The ICH formulates worldwide harmonization guidelines to guarantee uniform safety, effectiveness, and quality criteria for nanomedicines across various regulatory jurisdictions. Essential elements comprise standardized methodologies for the assessment of nanoparticle toxicity. Guidelines for ensuring uniformity and repeatability in nanomedicine formulations. Enhancing international collaboration in nanomedicine authorization to expedite clinical translation [205]. Effective communication of the dangers and advantages of nanomedicine is crucial for regulatory approval and public trust. As nanomedicine progresses in breast cancer therapy, ethical integrity, rigorous clinical studies, and regulatory uniformity will be essential for guaranteeing patient safety and broad accessibility. By tackling these obstacles, researchers and governments may promote responsible innovation and worldwide implementation of nanomedicine-based medicines.

## 6 Conclusion and Future Prospects

The intricate clinical manifestations and multifaceted origins of breast cancer continue to establish it as a predominant worldwide health concern. Although tumor treatment is becoming increasingly challenging, BC chemotherapy, as advanced nanotechnology, will remain helpful. This review seeks to elucidate the significance of pathophysiology and molecular subtypes of breast cancer, various risk factors with diagnosis, nanocarrier-based treatment approaches and the most recent findings in this area, with their limitations, nanotoxicity and ethical considerations are highlighted in the manuscript. Patents and promising clinical trials and regulatory aspects are also covered. It is now well acknowledged that only targeting traditional oncogenes in cancer cells is insufficient for treating all cancer patients. The majority of cancer patients benefit from medicines that target various components inside the tumor microenvironment, particularly immune cells, which are crucial for augmenting the body’s inherent antitumor immune response. Immunotherapeutics, similar to other pharmaceuticals, utilize nanotechnology to prolong their circulation half-lives, protect their integrity from circulating enzymes, and enhance their targeted delivery to tumors or tissues in patients. The successful clinical translation of nano-immune-therapeutics depends on several essential factors, including feasibility, toxicity, manufacturability, and cost-effectiveness. Despite extensive efforts to passively target different nanocarriers for breast cancer treatment, their anticancer effectiveness and accumulation in the targeted tissue remain constrained. Significantly, there have been considerable breakthroughs in molecular analysis methods in both preclinical and clinical environments, shown by the use of the serial measurements of molecular and architectural responses to treatment (SMMART) platform. The nanoparticles encapsulate genetic tools to safeguard them from degradation and augment their concentration in tumor cells, hence enhancing the efficiency of gene expression control and inhibiting breast cancer growth. Consequently, the simultaneous delivery of genes and pharmaceuticals can enhance breast cancer treatment. Despite the advancements in breast cancer treatment using gene therapy, its efficacy *in vivo* and in clinical trials is constrained by inadequate accumulation at the tumor site and the potential for enzymatic destruction in the bloodstream. Nanomaterials can be investigated in the delivery of genes for breast cancer suppression in the future, overcoming the associated limitations. To enhance the prevalence of nanoparticle-mediated drug delivery systems in clinical settings, a greater emphasis must be placed on therapeutic development and preclinical testing within the drug development process. Beyond lipid and basic polymeric nanoparticles, few additional nanoparticle-based therapeutics have successfully progressed to clinical assessment, despite encouraging preclinical results. This constraint mostly arises from the substantial development expenses and regulatory hurdles linked to intricate nanoparticle systems. Formulations with several components have challenges in elucidating processes, bioactivities, and toxicological characteristics. Moreover, they have difficulties pertaining to manufacturability (such as scalability and batch-to-batch repeatability) and stability, requiring adherence to stringent FDA regulatory standards. Moreover, to foster innovation and advancement in nanotechnology for breast cancer therapy, it is essential to establish interdisciplinary cooperation platforms that facilitate the seamless integration of scientific findings with clinical trials.

## Data Availability

Data sharing not applicable to this article as no datasets were generated or analyzed during the current study.
